# Maximized complexity in miniaturized brains: morphology and distribution of octopaminergic, dopaminergic and serotonergic neurons in the parasitic wasp, *Trichogramma evanescens*

**DOI:** 10.1007/s00441-017-2642-8

**Published:** 2017-06-09

**Authors:** Emma van der Woude, Hans M. Smid

**Affiliations:** Laboratory of Entomology, Wageningen University, P.O. Box 16, 6700 AA Wageningen, The Netherlands

**Keywords:** Monoamine, Confocal laser scanning, Immunohistochemistry, Miniaturization, Hymenoptera

## Abstract

The parasitic wasp, *Trichogramma evanescens*, is an extremely small insect, with a body length as small as 0.3 mm. To facilitate this miniaturization, their brains may have evolved to contain smaller neural components and/or reduced neural complexity than larger insects. Here, we study whether the size and number of neurons are reduced in the miniaturized brain of *T. evanescens*, focusing on neurons that express serotonin (5HT), octopamine (OA) and dopamine (DA). We provide the first description of the distribution, projection patterns and number of 5HT-, OA- and DA-like immunoreactive cell bodies in *T. evanescens* and compare our observations with descriptions of much larger insects. The brains of *T. evanescens* contain comparable numbers of monoaminergic neurons to those of larger insects. Serotonergic neurons appear to be especially conserved; most of the clusters contain a similar number of neurons to those described in *Apis mellifera* and *Drosophila melanogaster*. This maintained complexity may have been facilitated by miniaturization of neuron size. However, many dopaminergic and some octopaminergic neuron clusters in *T. evanescens* contain fewer neurons than in larger insects. Modification of the complexity of these monoaminergic systems may have been necessary to maintain neuron functionality during brain miniaturization in *T. evanescens*. Our results reveal some of the evolutionary adaptations that may enable behavioural and cognitive complexity with respect to miniaturized brains.

**Electronic supplementary material:**

The online version of this article (doi:10.1007/s00441-017-2642-8) contains supplementary material, which is available to authorized users.

## Introduction

The parasitic wasp,*Trichogramma evanescens* (Hymenoptera: Trichogrammatidae), is an extremely small gregarious parasitoid that lays its eggs inside the eggs of butterflies and moths. The adult body size of the wasps strongly depends, by means of phenotypic plasticity, on the level of nutrient availability inside the host egg. Genetically identical sister wasps reach body lengths as small as 0.3 mm when they develop in small host eggs or in competition with many developing larvae (Van der Woude et al. 2013) and can be as large as 0.9 mm when they develop in large hosts without competition from other wasp larvae (Van der Woude and Smid 2016).

*Trichogramma evanescens* show isometric brain scaling, exhibiting a linear relationship between brain and body volume. This deviates from the situation described by Haller’s rule, which states that small animals have relatively larger brains. As a result of brain isometry, the smallest *T. evanescens* have brains that are even smaller than that predicted by Haller’s rule. Their brain volume can be as small as 0.26*10^6^ μm^3^ (Van der Woude et al. 2013), which is almost 2500× times smaller than the brain of a honeybee (Mares et al. 2005).

Despite these extremely small brains, *Trichogramma* wasps can walk, fly, discriminate between odours and colours, live for several weeks and control the size, number and sex of their offspring (Suzuki et al. 1984; Waage and Ming 1984; Dutton and Bigler 1995; McDougall and Mills 1997; Pompanon et al. 1997; Keasar et al. 2000; Fatouros et al. 2008). Furthermore, they detect their host eggs by hitchhiking on butterflies that are ready to lay their eggs and learn to associate odours and colours with the presence of suitable hosts (Fatouros et al. 2005; Huigens et al. 2009). This indicates that strongly miniaturized brains can still generate a level of behavioural complexity and modulation that is, even in the smallest individuals, comparable with those of much larger insects.

Evolution of miniaturized brains could have resulted in reductions in the size of neural components, reductions in neural complexity or both. Indications of such modifications can be found by comparisons with larger species. For example, parasitic wasps of the genus *Cotesia* have body lengths that are 10-fold larger than that of *T. evanescens*. Depending on the size of the wasps, a 10– to 100-fold difference can be found in total volume of glomeruli inside the antennal lobes of the two wasps. However, only a 2-fold difference can be measured in antennal lobe complexity: *Cotesia* wasps have almost 200 glomeruli in the antennal lobe (Smid et al. 2003; Das and Fadamiro 2013), whereas *T. evanescens* wasps have 100 glomeruli (Van der Woude and Smid 2016).

Similar modifications may occur at the neuron level. Neuronal cell bodies and neurites are probably miniaturized as much as possible, within physical limits and further miniaturization can be achieved through the modifications of neuron number and arborization complexity. The physical limits of neuron size are determined by the minimum size that neurites need for adequate firing and that cell bodies need to contain their cell organelles. A decrease beyond these limits may severely affect the physical performance of neurons. Thinner axons, for example, have reduced neural firing frequencies and are more sensitive to the effects of the random opening and closing of ion channels (Faisal et al. 2005; Perge et al. 2012). A decrease of cell body volume affects the available space for cell organelles, of which the nucleus is the largest. Neuron performance may be affected when the size of the nucleus is reduced, because it might require a reduction of genome size (Gregory 2001) or even the formation of anucleate neurons (Polilov 2012).

To miniaturize brain size further, while avoiding the compromised performance of undersized neurons, the number of neurons and neuronal connections may need to be reduced. A reduction of neuron numbers can occur through a proportional reduction of neurons in all neural pathways or by removing some pathways entirely while maintaining others. For example, *Nasonia* parasitic wasps form fewer octopaminergic neurons in their brains than much larger honeybee workers (Sinakevitch et al. 2005; Haverkamp and Smid 2014). This lower number of neurons is attributable not only to the formation of fewer octopaminergic neurons in the neuron clusters that are present in both honeybees and *Nasonia* but also to the complete absence of some other clusters. Even more severe modifications of neuronal complexity may have been required to achieve even smaller brain sizes in *T. evanescens*.

In the present study, we investigate the way that the size and number of neurons are affected in the miniaturized brain of *T. evanescens*. We study quantifiable subpopulations of neurons that release serotonin (5HT), octopamine (OA) and dopamine (DA) as a neurotransmitter. The morphology and distribution of these neurons are well defined in a variety of insect species. This allowed us to compare the number, size and location of monoaminergic neurons in *T. evanescens* with those of larger hymenopterans, such as the parasitic wasps *Nasonia* and *Cotesia* (Bleeker et al. 2006; Haverkamp and Smid 2014) and the even larger honeybee (Schürmann and Klemm 1984; Schafer and Rehder 1989; Schürmann et al. 1989; Kreissl et al. 1994; Sinakevitch et al. 2005). It also allowed comparisons with the more distantly related but well-characterized, fruit fly (Monastirioti 1999; Sinakevitch and Strausfeld 2006; Busch et al. 2009; Mao and Davis 2009; Blenau and Thamm 2011). Serotonergic, octopaminergic and dopaminergic neurons are known to play critical roles in basic neural functioning. They are involved in a large variety of behavioural and physiological processes, including learning (Roeder 2005; Blenau and Thamm 2011; Burke et al. 2012; Yamamoto and Seto 2014). In the present study, we provide the first description of the distribution, projection patterns and number of 5HT-like immunoreactive (5HT-L-IR), OA-like immunoreactive (OA-L-IR) and DA-like immunoreactive (DA-L-IR) neurons in the miniaturized brain of *T. evanescens*. We also aim to find out whether the numbers of 5HT-L-IR, OA-L-IR and DA-L-IR neurons are smaller compared with those of larger insects.

## Materials and methods

### Insects

*Trichogramma evanescens* Westwood (Hymenoptera: Trichogrammatidae), inbred isofemale strain GD011, was reared in a climate room (22 ± 1 °C, 50–70% relative humidity, light:dark 16:8) by using hosts of different sizes, as previously described (Van der Woude et al. 2013; Van der Woude and Smid 2016). The body size of the wasps depends on the level of nutrient availability inside the host egg. Hence, use of differently sized hosts ensured that wasps emerged with body sizes within the entire natural range. We used host eggs of three species: small eggs of the Mediterranean flour moth *Ephestia kuehniella*, intermediate-sized eggs of the cabbage moth *Mamestra brassicae* and large eggs of the tobacco hornworm *Manduca sexta*. From the wasps that emerged from these hosts, we randomly selected individuals of a large variety of body sizes for our experiments, in order to ensure that the entire natural range of body sizes was represented by our study. Eggs of *E. kuehniella* were obtained as UV-irradiated eggs from Koppert Biological Systems (Berkel en Rodenrijs, The Netherlands). *Mamestra brassicae* were reared on cabbage plants (*Brassicae oleracea*) in a climate room (21 ± 2 °C, 50–70% relative humidity, light:dark 16:8). Adult moths oviposited on filter paper and their eggs were used fresh for rearing procedures. *Manduca sexta* were obtained as pupae from the Max Planck Institute for Chemical Ecology (Jena, Germany) and kept in a flight cage with tobacco plants (*Nicotiana tabacum* SR1) inside a climate cabinet (25 ± 1 °C, light:dark 16:8). Eggs were collected from the plants and frozen until used in rearing procedures.

### Analysis of 5HT immunoreactivity

Two-day-old female *T. evanescens* (body lengths ranging between 0.3 and 0.9 mm) were immersed in ice-cold 4% formaldehyde in 0.1 M phosphate buffer (pH = 7.2), freshly prepared from paraformaldehyde (Merck, Darmstadt, Germany). The wasps were subsequently decapitated, their antennae were removed and the eyes were carefully opened with fine tweezers (Dumont no. 5, Sigma-Aldrich) to allow optimal infiltration of the fixative. Heads were fixed either for 4 h at room temperature or overnight at 4 °C and subsequently rinsed in four changes of phosphate-buffered saline (PBS; Oxoid, Dulbecco “A” tablets). Access to the brain for further procedures and microscopic analysis was achieved by removing either the anterior or posterior cuticle with fine tweezers in PBS at room temperature. Permeability of the tissue was improved by incubating heads in 0.05% collagenase (Sigma-Aldrich) in PBS for 45 min at room temperature, followed by rinses (4 × 10 min) in 0.5% Triton X-100 in PBS (PBS-T). The heads were then pre-incubated for 1 h in 10% normal goat serum (NGS; Dako, Denmark) in PBS-T (PBS-T-NGS) and subsequently incubated in a 1:200 dilution of rabbit anti-5HT antibodies (Millipore catalogue no. AB938, RRID:AB_92263) in PBS-T-NGS overnight at room temperature. After being rinsed (6 × 30 min) in PBS-T at room temperature, the heads were incubated in a secondary antiserum of goat-anti-rabbit antibodies linked to Alexa Fluor 488 (Molecular Probes, catalogue no. A11008, RRID:AB_143165) at a dilution of 1:200 and in propidium iodide (Sigma-Aldrich) at a dilution of 1:500 in PBS-T-NGS for 4 h at room temperature. The heads were then rinsed in PBS-T (4 × 30 min) and in PBS (4 × 30 min), dehydrated in a graded series of ethanol (30–50–70-90-96-100-100%, 2 min each) and cleared in xylene. Finally, the heads were mounted in DPX (Sigma-Aldrich) with the opened side of the head facing the cover slide.

### Analysis of OA immunoreactivity

Two-day-old female *T. evanescens* (body lengths ranging between 0.3 and 0.9 mm) were given an oviposition experience on fresh *M. brassicae* eggs 30 min before dissection. Such an oviposition experience has previously been shown to increase immunolabelling in *Nasonia* parasitic wasps (Haverkamp and Smid 2014). Ovipositing wasps were removed from the host eggs by their wings by using fine tweezers. The wasps were directly placed in a dissection tray containing fixative, which consisted of three parts saturated picric acid, one part 25% glutaraldehyde (Sigma-Aldrich) and one part 0.1% acetic acid, at room temperature. The head capsule was opened to allow the infiltration of the fixative and the heads were subsequently fixed at room temperature for 4 h or overnight.

After fixation, heads were rinsed in several changes of 70% ethanol, after which either the anterior or posterior cuticle was removed with fine tweezers. The heads were subsequently dehydrated by using a graded series of ethanol (30–50–70-90-96-100-100%, 2 min each), degreased in xylene for 20 s and rehydrated with the same graded series in reverse order back to PBS. Oxidization of OA was reduced by treatment in 0.1% or 1% sodium borohydride (Sigma-Aldrich) in PBS for 20 min, followed by four changes of PBS. A treatment of 0.05% collagenase in PBS (45 min at room temperature) was used to increase the permeability of the tissue. This was followed by rinses (4 × 5 min) in PBS-T and pre-incubation (1 h) in PBS-T-NGS. The heads were then incubated in a 1:200 dilution of rabbit anti-OA antibodies (MoBiTec, catalogue no. 1003GE, RRID:AB_2314999) in PBS-T-NGS. After further rinses (6 × 30 min) in PBS-T, a secondary antiserum of goat-anti-rabbit antibodies linked to Alexa Fluor 488 (Molecular Probes, catalogue no. A11008, RRID:AB_143165) was used at a dilution of 1:200 in PBS-T-NGS together with propidium iodide at a dilution of 1:500. Heads were then further processed as described above for mounting in DPX.

### Analysis of DA immunoreactivity

Immunohistochemical procedures for dopamine analysis were similar to those for octopamine analysis, except that the wasps did not receive an oviposition experience prior to dissection. The wasps were directly placed in the fixative and, after the opening of the cuticle, all heads were fixed for 3 h at room temperature. Further processing was identical to that described above, but with mouse anti-DA (Millipore, catalogue no. MAB5300, RRID:AB_94817) as primary antibody at a dilution of 1:200 in PBS-T-NGS overnight at room temperature. After several rinses (6 × 30 min) in PBS-T, a secondary antibody of rabbit-anti-mouse (Dako, catalogue no. Z0259, RRID:AB_2532147) was applied at a 1:200 dilution for 3 h at room temperature. Finally, a tertiary antiserum of goat-anti-rabbit antibodies linked to Alexa Fluor 488 (Jackson ImmunoResearch Labs, catalogue no. 115–545-003, RRID:AB_2338840) was used at a dilution of 1:200 together with propidium iodide at a dilution of 1:500 overnight at 4 °C. Heads were then further processed as described above for mounting in DPX.

### Antisera specificity

The specificity of the rabbit anti-serotonin antibody was provided by the manufacturer (Mobitec, Germany). Evaluation of the antisera showed positive immunofluorescence in serotonin-containing human ileum structures. The specificity of the rabbit anti-octopamine antibody was determined as specified by the manufacturer (Mobitec, Germany) by using conjugated octopamine-glutaraldehyde proteins: OA-G-BSA 1; Noradrenaline-G-BSA 1:90; Tyramine-G-BSA 1:142; L-DOPA-G-BSA 1:285; OA=G=BSA 1:442; DA-G-BSA 1:1120; Adrenaline-G-BSA 1:>10,000; OA 1:>10,000. Cross-reactivity of the mouse anti-dopamine antibody was determined as specified by the manufacturer (Mobitec, Germany): DA-G-BSA 1; L-DOPA-G-BSA 1:10,000; Tyrosine-G-BSA 1:36,000; Tyramine-G-BSA 1:>50,000; Noradrenaline-G-BSA 1:>50,000; OA-G-BSA 1:>50,000; Adrenaline-G-BSA 1:>50,000; DA 1:>50,000. We performed additional control experiments by using preparations without primary antisera. These did not reveal any immunolabelling.

### Microscopy

A Zeiss LSM 510 confocal laser scanning microscope with a 488-nm argon laser was used with a band-pass emission filter at 505–550 nm to visualize Alexa Fluor 488 and a long-pass emission filter at 560 nm for propidium iodide. Heads were scanned by using a Plan-Apochromat ×63 oil immersion objective (N.A. 1.4). The resolution was kept at 1024 × 1024 pixels and 8 bit. Voxel size ranged between 0.14 × 0.14 × 0.70 μm for overview scans of whole brains and 0.07 × 0.07 × 0.20 μm for detailed scans of cell clusters. We did not correct for Z-axis refractive index mismatch, because the refractive index of the employed immersion oil matched the index of the mounting medium.

### Orientation and nomenclature

The head of *T. evanescens* has a vertical orientation with ventral mouthparts. The orientations that were used in this study to indicate locations inside the brain, therefore, refer to the position along the anterior-posterior body axis. To identify clusters, we followed nomenclature as described for OA-L-IR neurons in the parasitic wasps *Nasonia vitripennis* and *Nasonia giraulti* (Haverkamp and Smid 2014). In this system, cell clusters were numbered from anterior to posterior. A similar nomenclature system was previously used for *Apis mellifera* (Schürmann and Klemm 1984; Sinakevitch et al. 2005). We followed the numbering of corresponding clusters in previous studies, where possible but deviated from these descriptions when clusters appeared in a different order.

We based our description of the location and projection of neurons on the general morphology of brain compartments as described for *A. mellifera* (Brandt et al. 2005) and *Nasonia* (Haverkamp and Smid 2014). To identify corresponding areas in *T. evanescens*, we used the propidium iodide and background staining in preparations of the present study, in combination with previous preparations stained with the neuropil marker, mouse monoclonal antibody nc82 (Van der Woude and Smid 2016). The general morphology of brain compartments in *T. evanescens* corresponded to the descriptions of *Nasonia* (Haverkamp and Smid 2014) and *A. mellifera* (Brandt et al. 2005) with three exceptions. First, only a single mushroom body calyx was visible in *T. evanescens*, whereas *Nasonia* and *A. mellifera* both had elaborate double calyces. The formation of single calyces is not uncommon among wasps of the superfamily Chalcidoidea (which includes *Trichogramma* but also *Nasonia*) and has been suggested to be the consequence of miniaturization (Farris and Schulmeister 2011). Second, no clear transition of the sub- into the supraoesophageal zone was seen. Hence, we do not distinguish between these two zones and define “brain” as the combination of the sub- and supraoesophageal zones. Third, we could not observe the distinction of the mandibular, maxillary and labial neuromeres in the suboesophageal zone of *T. evanescens*. This complicated the nomenclature of the octopaminergic ventral unpaired median neurons (OA-VUM). These neurons are located in the midline of the suboesophageal zone in various insect species and are usually named after the neuromere in which they occur (Schroter et al. 2007; Haverkamp and Smid 2014). The OA-VUM cell bodies were located very close together in *T. evanescens*. We therefore combined them all into one cluster: OA-VUM.

### Neuron analysis

We selected the 30 best-stained brains per monoamine analysis for cell body counts. The diameter and number of cell bodies were only analysed in brains in which the cluster of interest was clearly visible and the best-stained hemisphere was selected for analysis of the cluster. To count cell bodies that were located close together, we used image segmentation manually to trace cell bodies. We used either the segmentation editor of Amira 5.4 (Visage Imaging, Berlin, Germany) or the TrakEM2 plugin (Cardona et al. 2012) in the Fiji package of ImageJ 1.50c (Schindelin et al. 2012). Cell body diameters were measured with the measuring tool in the Fiji package of ImageJ. Each cell was measured twice and measurements of all cells within a cluster were averaged to obtain a single average value per cluster per brain. The measuring tool was also used to measure brain width, which was measured from medulla to medulla to avoid lamina areas that were damaged by the dissection procedures.

We included brains within the entire natural size range in our analysis. The size of the wasps did not affect the distribution and number of monoaminergic neurons in the brain but an effect was detectable on neuron diameter (Van der Woude and Smid 2017). Hence, we presently report the average diameter of cell bodies from the total body size range to cover natural variation. Descriptions of neuron projection patterns were prepared from those preparations in which they were best visible, which were mostly large brains. We used the z-project function in the Fiji package of ImageJ 1.50c to create z-stack projections of cell bodies and neurites. The contrast of these images was enhanced in Adobe Photoshop CS6 (San Jose, Calif., USA).

## Results

### Overall quality of immunolabelling

All antisera yielded good staining qualities and revealed many neuron clusters but the 5HT-L-IR staining was more intense than the OA- and DA-L-IR staining. Differences were visible neither in the staining quality between brains of different sizes nor in the number or distribution of the monoaminergic neuronal cell bodies. However, neurites were more visible in large than in small brains because of their larger diameter and length. We will further compare the specific differences between small and large sister wasps in a different study (Van der Woude and Smid 2017).

The average brain width (measured from medulla to medulla) was 136 ± 30 μm (*n* = 30) in wasps that were analysed for 5HT-like immunoreactivity, 123 ± 19 μm (*n* = 30) in wasps that were analysed for DA-like immunoreactivity and 125 ± 18 μm (*n* = 30) in wasps that were analysed for OA-like immunoreactivity. Although the dissection of brains of such small sizes was possible without severe damages to neuropil tissue, our methods caused some difficulties. Our method of dissecting the brains after tissue fixation made the brains less fragile and therefore easier to separate from the cuticle but also reduced tissue elasticity. Three specific areas were rather vulnerable to subsequent tissue damage during the dissection procedures. First, the ventral rim of the brain was sometimes damaged because of its tight attachment to the inflexible area close to the mouthparts. This may have influenced our analysis of the clusters that are located in the ventro-medial brain area, such as OA-VUM and DA-4. Second, the lamina was often damaged because of its close attachment to the retina, which had to be removed for laser penetration during imaging procedures. We therefore only included descriptions of lamina innervation from preparations in which this area was not damaged and excluded the laminas in our estimations of brain width. Third, the area around the oesophageal foramen was often damaged during decapitation, when the connection between the oesophagus and the remaining digestive tract was severed. This may have caused variation in our analysis of cluster OA-3 in this area.

### Distribution of 5HT-L-IR neurons

**Fig. 1:**
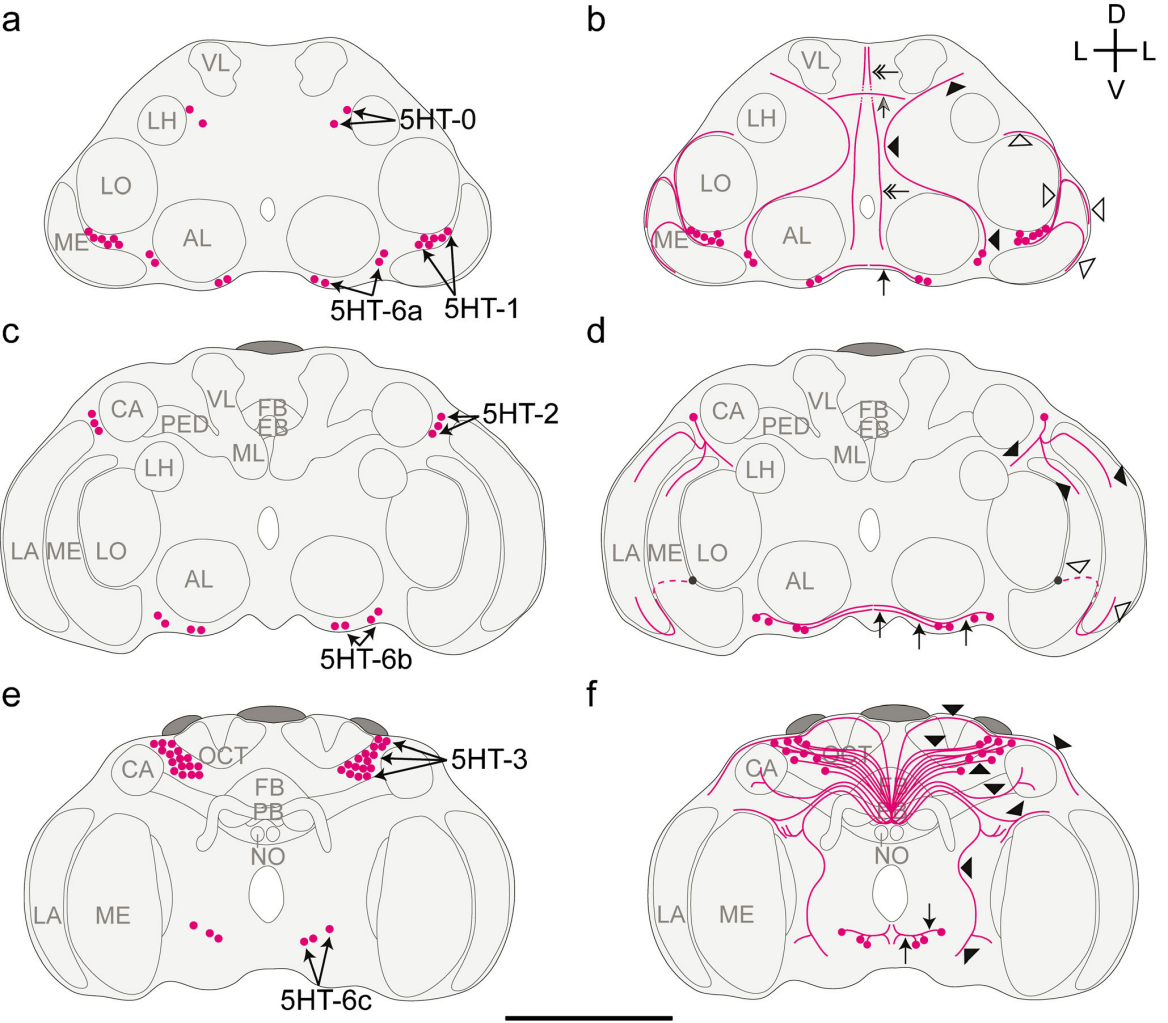
Representation of the location of serotonin-like immunoreactive (5HT-L-IR) cell bodies (**a**, **c**, **e**) and projections (**b**, **d**, **f**) in the brain of *Trichogramma evanescens* shown dorsal side up at three locations through the depth of the brain. **a**, **b** Anterior view at approximately one quarter through the depth of the brain. **a** Location of clusters 5HT-0, 5HT-1 and 5HT-6a. **b** The most anterior neurite (*grey arrow*) projects in a lateral direction from the brain midline. Slightly more posteriorly, a neurite runs in a dorsal direction along the brain midline (*feathered arrows*). The neurites of the 5HT-1 cluster (*open arrowheads*) project along the lateral rims of the lobula and medulla. The 5HT-6a neurons on the lateral side of the antennal lobe project in a dorso-posterior direction (*black arrowheads*) following the brain midline before bending in a more lateral direction towards the dorso-lateral neuropil rim. Neurites of the medial 5HT-6a neurons (*black arrow*) form a network of fine bifurcations and varicose terminals in the ventro-posterior part of the brain. **c**, **d** Anterior view halfway through the depth of the brain. **c** Location of clusters 5HT-2 and 5HT-6b. **d** The neurites of 5HT-2 (*black arrowheads*) innervate the medulla and dorso-posterior side of the lamina. The ventro-anterior side of the lamina is innervated by a bifurcation of 5HT-1 (*open arrowheads*) that projects from the cluster shown in **b** (*grey cell body* and *dashed line* show continuation from **b**). Neurites of 5HT-6b (*black arrows*) join the network of bifurcations and varicose terminals formed by 5HT-6a. **e**, **f** Posterior view three quarters through the depth of the brain. **e** Location of clusters 5HT-3 and 5HT-6c. **f** The projections from the 5HT-3 cell bodies (*arrowheads*) innervate most neuropil areas. Primary neurites project towards the brain midline and form a dense network. Neurites from this network project in lateral, dorso-anterior and ventro-anterior directions, innervating the mushroom body pedunculus and calyx and projecting towards the optic lobes and antennal lobes. Neurites of 5HT-6c (*arrows*) join the network of bifurcations and varicose terminals formed by 5HT-6a and 5HT-6b. Bifurcations also project anteriorly and posteriorly (*AL* antennal lobe, *LA* lamina, *ME* medulla, *LO* lobula, *LH* lateral horn, *CA* calyx, *PED* pedunculus, *VL* vertical lobe, *ML* medial lobe, *FB* fan-shaped body, *EB* ellipsoid body, *PB* protocerebral bridge, *NO* noduli, *OCT* ocellar tract, *D* dorsal, *V* ventral, *L* lateral). *Bar* 50 μm (average-sized brain)

The 5HT-L-IR staining was very intense and revealed many 5HT-L-IR neuron clusters and neurites (Fig. 1). The average diameter of 5HT-L-IR cell bodies was 2.1 ± 0.44 μm (*n* = 175). Neurites were approximately 0.5 μm in diameter and varicose terminals were approximately 1 μm in diameter.

**Fig. 2:**
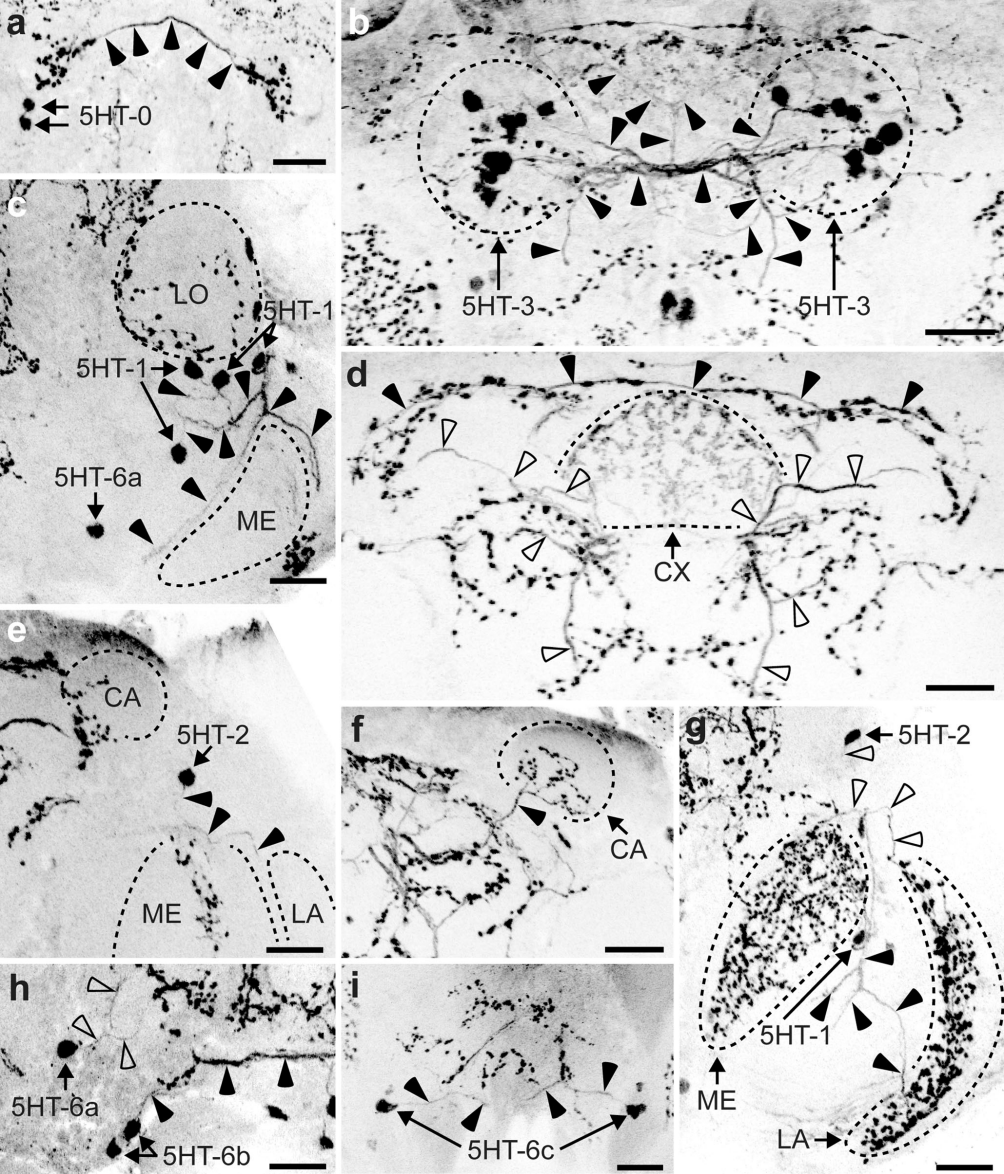
Z-stack projections of confocal images showing serotonin-like immunoreactive (5HT-L-IR) cell bodies and projection patterns in *T. evanescens*. **a** Cell cluster 5HT-0 is the most anterior cluster of two neuron pairs (*arrows*) located directly beneath the anterior cuticle (*arrowheads* the most anterior neurite that projects in a lateral direction from the brain midline and gives off varicose terminals dorsal to 5HT-0 cell bodies). **b** The most prominent cell cluster of up to 16 neuron pairs in *T. evanescens* is 5HT-3 (*arrows*). The neurites (*arrowheads*) project medially and form a network of bifurcations (as indicated in Fig. 1f) that project anteriorly and innervate most of the central brain. **c** Cell cluster 5HT-1 is located anteriorly between the lobula (*LO*) and medulla (*ME*) and consists of up to six neuron pairs (*arrows*). Neurites (*arrowheads*) from this cluster bifurcate, project along the rim of the medulla and innervate the lamina. **d** Overview of the posterior central brain showing the innervation of the central complex (*CX*), neurites that run along the dorsal rim of the brain (*black arrowheads*) and neurites from the 5HT-3 network (*open arrowheads*, also shown in **b**). **e** The single neuron of the cell cluster 5HT-2 (*arrow*) is located on the lateral side of the mushroom body calyx (*CA*) and its neurites (*arrowheads*) innervate the optic lobes. **f** Innervation of the mushroom body calyx (*CA*) by a neurite of the 5HT-3 network (*arrowhead*). **g** Overview of the innervation of the medulla (*ME*) and lamina (*LA*), which contain a layered pattern of varicose terminals originating from bifurcations of 5HT-1 (*black arrowheads*, also shown in **c**) and 5HT-2 (*open arrowheads*, also shown in **e**). **h** Neurons of cluster 5HT-6b (up to four neuron pairs, *arrows*) in the ventral rim of the brain project towards the brain midline (*black arrowheads*). The lateral 5HT-6a neuron (up to four neuron pairs, *arrow*) projects in dorsal direction (*open arrowheads*). **i** The most posterior cell cluster of up to three neuron pairs is 5HT-6c (*arrow*). Neurites (*arrowheads*) project medially and join the network of bifurcations of 5HT-6a and 5HT-6b neurons (*D* dorsal, *V* ventral, *A* anterior, *P* posterior). Figures are oriented with the dorsal side pointing upwards. Contrast of z-stack projections was enhanced in Adobe Photoshop CS6 (San Jose, Calif., USA). *Bars* 10 μm

Cluster 5HT-0 (Fig. 2a) is the most anterior serotonergic cell cluster in *T. evanescens*, being located directly underneath the frontal cuticle and dorsal to the lobula. We indicate these cell bodies as 5HT-0 because they do not correspond to any of the clusters that are present in *A. mellifera* and because they are located more anteriorly than 5HT-1 (Schürmann and Klemm 1984). The close location to the cuticle resulted in damage of this cluster when the anterior head cuticle was removed. We therefore only analysed this cluster in heads from which the posterior cuticle had been removed. Cluster 5HT-0 invariably consisted of two pairs of neurons, with an average diameter of 2.0 ± 0.33 μm (*n* = 6). The primary neurites of this cluster were not visible.

Cluster 5HT-1 (Fig. 2c) is located ventro-laterally to the anterior side of the lobula and innervates the optic lobes in the same hemisphere. This cluster contains up to six pairs of neurons (on average 4.5 ± 1.04 pairs), with an average diameter of 2.0 ± 0.38 μm (*n* = 30).

Cluster 5HT-2 (Fig. 2e) is located laterally to the mushroom body calyx and contains only a single pair of serotonergic neurons in most preparations. In two preparations, however, two and three pairs were found, respectively, in this cluster. This results in an average count of 1.1 ± 0.40 neurons per cluster, with an average diameter of 2.1 ± 0.39 μm (*n* = 30).

Cluster 5HT-3 (Fig. 2b) is the most pronounced group of serotonergic cell bodies in *T. evanescens*. These neurons are located posteriorly and medially to the calyx of the mushroom body and their position is lateral to the ocellar tract, close to the posterior cuticle. They are always well-stained and innervate a large part of the anterior neuropil. We counted up to 16 neuron pairs in this cluster (on average 12.1 ± 1.70 pairs; *n* = 21), with an average diameter of 2.1 ± 0.48 μm (*n* = 21).

The cell clusters that were described as 5HT-4 and 5HT-5 in *A. mellifera* (Schürmann and Klemm 1984) were not observed in *T. evanescens*.

Three clusters of 5HT-L-IR cell bodies are present at the ventral rim of the brain, directly ventro-posterior to the antennal lobe and further posteriorly. Their location corresponds to the location of 5HT-6 neurons in *A. mellifera* (Schürmann and Klemm 1984), which are located in the labial, maxillary and mandibular neuromeres of the suboesophageal zone (Seidel and Bicker 1996). We could distinguish between three subclusters and named them 5HT-6a, 5HT-6b and 5HT-6c, from anterior to posterior.

The 5HT-6a neurons (Fig. 2h) lie directly ventro-posterior to the antennal lobes, where they can be found both on the lateral and medial side of the antennal lobe. We counted up to four pairs of neurons in this cluster (on average 2.3 ± 1.05; *n* = 30), with an average diameter of 2.2 ± 0.48 μm. Cluster 5HT-6b (Fig. 2h) lies in the ventral rim of the brain, approximately central in the depth of the brain. We counted up to four pairs of neurons in this cluster (on average 2.1 ± 0.99; *n* = 30), with an average diameter of 2.1 ± 0.41 μm. Cluster 5HT-6c (Fig. 2i) is the most posterior cluster in the ventral rim of the brain. We counted up to three pairs of neurons in this cluster (on average 2.1 ± 0.89; *n* = 28), with an average diameter of 2.2 ± 0.50 μm.

### Projection patterns of 5HT-L-IR neurons

Although 5HT-L-IR neurites are thin (approximately 0.5 μm in diameter), the primary neurites of 5HT-L-IR cell bodies could be traced for all clusters, except for 5HT-0. The most anterior neurite projects in a lateral direction from the brain midline and gives off varicose terminals dorsal to 5HT-0 cell bodies (Fig. 2a). Just posteriorly, a neurite runs in a dorsal direction along the brain midline. These anterior neurites could not be traced further and so their origin and destination remain unknown.

The neurites that originate from neurons in clusters 5HT-1 and 5HT-2 innervate the optic lobes. The primary neurites of cluster 5HT-1 (Fig. 2c, g) project in a dorso-posterior direction along the lateral rim of the lobula and bifurcate at the edge of the medulla. One bifurcation continues along the lateral rim of the lobula in a dorso-posterior direction. Two other bifurcations run along the lateral rim of the medulla: one in a ventro-anterior direction and one in a ventro-posterior direction. The latter bifurcation innervates the ventro-anterior side of the lamina.

The primary neurite of cluster 5HT-2 (Fig. 2e, g) projects in a ventral direction towards the optic lobes, where it bifurcates into three neurites. One of these continues in a ventro-anterior direction along the lateral rim of the medulla. It gives off a dense network of varicose terminals on the posterior side of the medulla. Another bifurcation projects in a ventro-posterior direction along the medial rim of the lobula but could not be traced further. A third bifurcation continues in a ventro-posterior direction along the lateral rim of the medulla and innervates the dorso-posterior side of the lamina. Together with the 5HT-1 bifurcation that innervates the ventro-anterior side of the lamina, it forms a single layer of varicose terminals parallel to the surface of the eye. Compared with the lamina and medulla, the innervation of the lobula is sparse and consists of weakly labelled varicose terminals without a clear pattern. The origin of this innervation could not be traced.

The projections that originate from cluster 5HT-3 (Fig. 2b, d) are very prominent in *T. evanescens*. These neurites appear to innervate most neuropil areas. The primary 5HT-3 neurites project towards the brain midline, where they form a dense network. Neurites from this network project in lateral, dorso-anterior and ventro-anterior directions. The dorso-anteriorly projecting neurites follow the dorsal neuropil rim in the direction of the optic lobes. The neurite that projects laterally enters the mushroom bodies through the pedunculus and projects towards the calyx, where it bifurcates and gives off many varicose terminals (Fig. 2f). The most pronounced neurite from the 5HT-3 neurite network runs in a ventro-anterior direction (Fig. 1f) and bifurcates close to the dorsal rim of the medulla. One bifurcation continues in the direction of the medulla but could not be traced further. The other projects ventro-anteriorly in the direction of the posterior side of the antennal lobe, where it bifurcates again. These bifurcations could not be traced further. A second ventro-anteriorly projecting neurite from the 5HT-3 neurite network projects in the direction of the medulla, where it bifurcates on the dorsal side of the medulla. The bifurcations then run along the lateral and medial medulla rim but could not be traced further.

The central brain contains many small varicose terminals, which appear to originate mainly at neurites of the 5HT-3 network. The only neuropil area that is completely devoid of any 5HT immunoreactivity is the antennal lobe. Within the central complex, the ellipsoid body and fan-shaped body are clearly visible, because they are richly innervated (Fig. 2d), in contrast to the protocerebral bridge and noduli. The origin of the innervation of the central complex could not be traced.

The 5HT-6a neurons on the lateral side of the antennal lobe project in a dorso-posterior direction, following the brain midline before bending in a more lateral direction towards the dorso-lateral neuropil rim. Neurites of the medial 5HT-6a neurons join the neurites of 5HT-6b and 5HT-6c and together form a network of fine bifurcations and varicose terminals in the ventro-posterior part of the brain (Fig. 2h). In addition to contributing to this network, the 5HT-6c neurons also bifurcate into a posteriorly and an anteriorly projecting neurite. The posteriorly projecting neurite possibly descends to the thoracic ganglia but we did not study this. The anteriorly projecting neurite could not be traced further.

### Distribution of OA-L-IR neurons

**Fig. 3:**
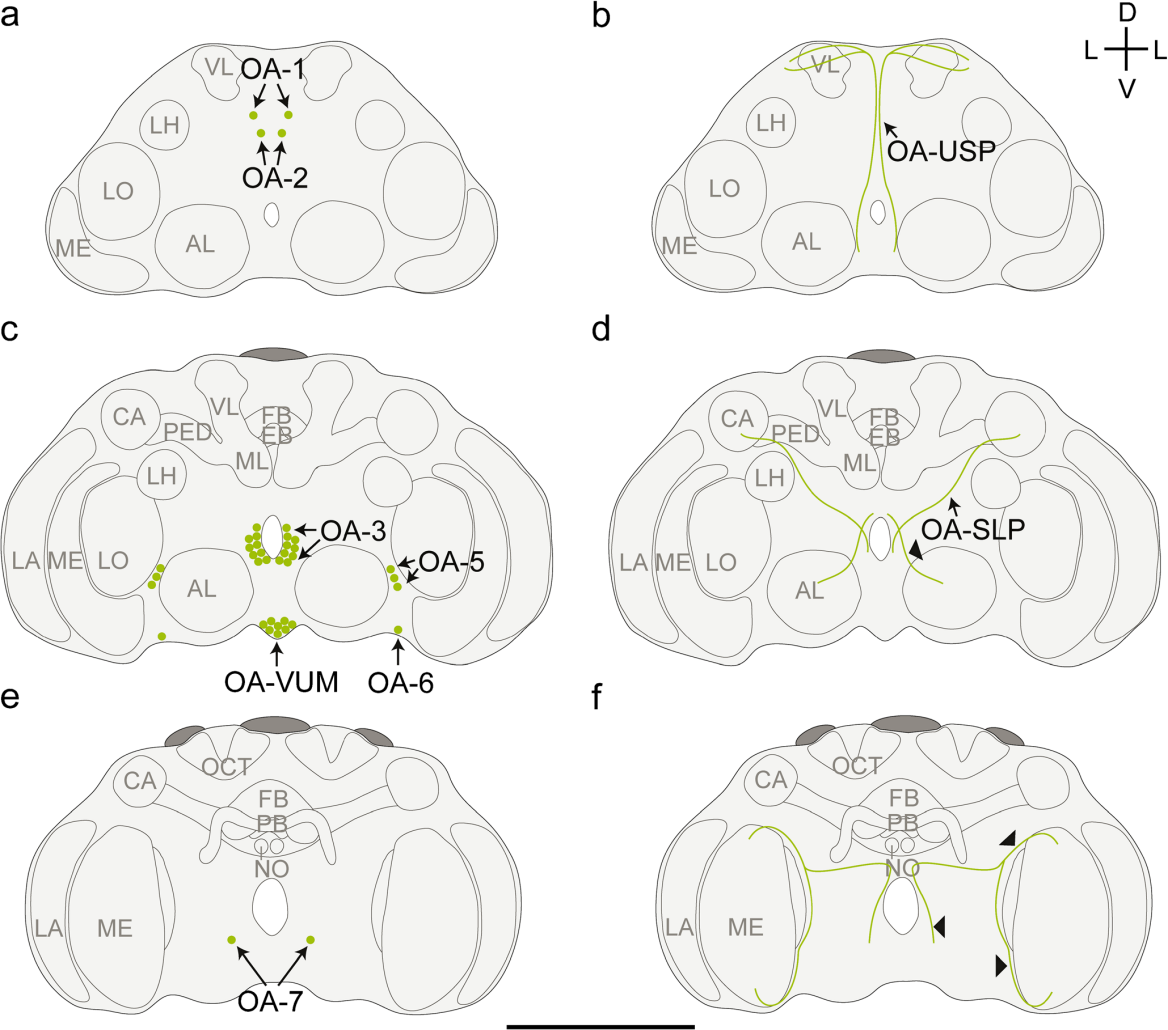
Representation of the location of octopamine-like immunoreactive (OA-L-IR) cell bodies (**a**, **c**, **e**) and projections (**b**, **d**, **f**) in the brain of *T. evanescens*, shown dorsal side up at three locations through the depth of the brain. **a**, **b** Anterior view at approximately one quarter through the depth of the brain. **a** Location of clusters OA-1 and OA-2. **b** The umbrella-shaped projection (OA-USP, *arrow*) originates at the ventro-medial side of the brain and runs in a dorsal direction along the brain midline. It passes the posterior side of the OA-3 cluster and bifurcates at the dorso-posterior side of the brain. **c**, **d** Anterior view halfway through the depth of the brain. **c** Location of clusters OA-3, OA-5, OA-6 and OA-VUM (ventral unpaired median neurons). **d** Several neurites form a network that surrounds the oesophageal foramen. The most anterior neurite from this network (*arrowhead*) projects laterally and innervates the antennal lobe. The stag-like projection (OA-SLP, *arrow*) projects in a dorso-lateral direction and innervates the mushroom body calyx. **e**, **f** Posterior view three quarters through the depth of the brain. **e** Location of cluster OA-7. **f** The optic lobes are innervated by a posterior neurite (*arrowheads*) that bifurcates into a neurite that innervates the dorso-posterior side of the medulla and by a neurite that projects anteriorly along the medial rim of the lobula and innervates the ventro-anterior side of the medulla (*AL* antennal lobe, *LA* lamina, *ME* medulla, *LO* lobula, *LH* lateral horn, *CA* calyx, *PED* pedunculus, *VL* vertical lobe, *ML* medial lobe, *FB* fan-shaped body, *EB* ellipsoid body, *PB* protocerebral bridge, *NO* noduli, *OCT* ocellar tract, *D* dorsal, *V* ventral, *L* lateral). *Bar* 50 μm (for an average-sized brain)

The OA-L-IR staining was clear but less intense than the 5HT-L-IR staining. The staining revealed many OA-L-IR neuron clusters and several neurites (Fig. 3). The average diameter of the OA-L-IR cell bodies was 3.3 ± 0.75 μm (*n* = 88). Neurites and varicose terminals had average diameters of approximately 0.6 μm.

**Fig. 4:**
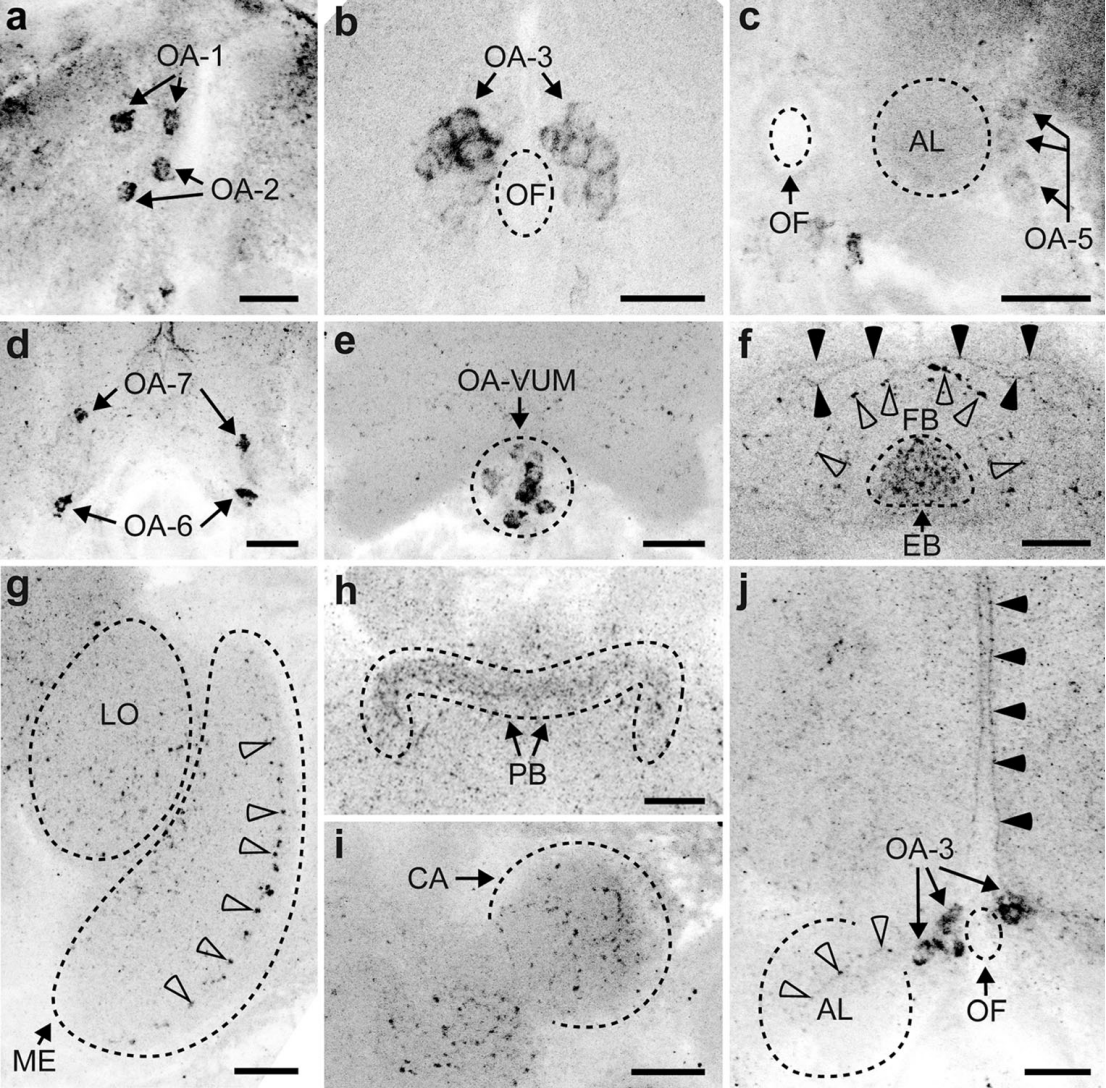
Z-stack projections of confocal images showing octopamine-like immunoreactive (OA-L-IR) cell bodies and projection patterns in *T. evanescens*. **a** OA-1 consists of a single pair of neurons (*arrows*) and is located directly beneath the anterior cuticle, close to the brain midline. Cluster OA-2 also consists of a single pair of neurons (*arrows*) and is located directly ventro-posterior to OA-1. **b** Cluster OA-3 (*arrows*, also shown in **j**) consists of up to nine neuron pairs and is located directly adjacent to the oesophageal foramen (*OF*). **c** Cluster OA-5 (*arrows*) consists of up to three neuron pairs and is located latero-posteriorly to the antennal lobe. **d** Cluster OA-6 (*arrows*) contains a single neuron pair at the lateral part of the ventral rim of the brain, whereas cluster OA-7 (*arrows*) is located more posteriorly ventro-lateral to the oesophageal foramen and also contains a single neuron pair. **e** Cluster OA-VUM (*arrows*) lies at the ventro-medial base of the brain and contains up to 13 unpaired neurons. **f** An arch of varicose terminals (*open arrowheads*) outlines the dorsal rim of the fan-shaped body (*FB*). The ellipsoid body (*EB*) contains a high density of varicose terminals. Dorsal to the fan-shaped body, the umbrella-shaped projection (OA-USP, *black arrowheads*) bifurcates and projects laterally. **g** OA-like immunoreactivity in the lobula (*LO*) and medulla (*ME*). *Arrowheads* show a layer of varicose terminals in the medulla. **h** The protocerebral bridge (PB) is clearly visible because of the high density of varicose terminals. **i** The mushroom body calyx (*CA*) contains several OA-L-IR varicose terminals. **j** Cluster OA-3 (*arrows*, also shown in **b**) may contribute neurites to the neurite network around the oesophageal foramen (*OF*). A neurite of this network projects towards the antennal lobes (*AL*, *open arrowheads*). The umbrella-shaped projection (OA-USP, *black arrowheads*) originates at the ventral side of the brain and projects in a dorsal direction along the brain midline. Figures are oriented with the dorsal side pointing upwards. Contrast of z-stack projections was enhanced in Adobe Photoshop CS6. *Bars* 10 μm

Cluster OA-1 (Fig. 4a) is the most anterior cluster of the OA-L-IR neurons. It consists of a single pair of cell bodies, located close to the anterior cuticle with an average diameter of 3.9 ± 0.91 μm (*n* = 9). Neurons of this pair are approximately 6–10 μm apart from each other.

Cluster OA-2 (Fig. 4a) also consists of a single pair of OA-L-IR neurons, located directly posterior and ventral to cluster OA-1. Cell bodies of this pair are slightly closer together (approximately 3–7 μm). The average diameter of these cells was 3.9 ± 0.76 μm (*n* = 9).

Cluster OA-3 (Fig. 4b) is the most pronounced OA-L-IR neuron cluster in *T. evanescens*. It is located ventro-posteriorly to cluster OA-2 and directly adjacent to the oesophageal foramen. We counted up to nine neuron pairs (on average 4.7 ± 1.61; *n* = 22), with an average diameter of 3.1 ± 0.66 μm (*n* = 22).

The OA-L-IR cluster in the dorsal rim of the brain, which has been described as OA-4 in *A. mellifera* (Sinakevitch et al. 2005), *Drosophila melanogaster* (Sinakevitch and Strausfeld 2006) and *N. vitripennis* (Haverkamp and Smid 2014), was not observed in *T. evanescens*.

Cluster OA-5 (Fig. 4c) is located latero-posteriorly to the antennal lobe. We found up to three pairs of OA-L-IR cell bodies in this cluster (on average 2.1 ± 0.83), with an average diameter of 3.1 ± 0.68 μm (*n* = 14).

Cluster OA-6 (Fig. 4d) consists of a single neuron pair in the ventro-lateral rim of the brain, ventro-posterior to the antennal lobes and medial to the medulla. The average diameter of these cells was 3.5 ± 0.74 μm (*n* = 11).

Cluster OA-7 (Fig. 4d) is the most posterior OA-L-IR neuron cluster, located ventro-laterally to the oesophageal foramen and close to the posterior cuticle. It consists of a single pair of neurons, which are also the smallest octopaminergic neurons with an average diameter of 2.8 ± 0.35 μm (*n* = 5).

The OA-VUM neurons (Fig. 4e) in *T. evanescens* lie at the ventral base of the brain, very close to the mouthparts. This location is vulnerable to damage caused by the dissection procedure, as described above. We counted up to 13 OA-VUM neurons in two exceptionally well-stained preparations but, on average, only 4.4 ± 3.36 neurons were visible (*n* = 18) with an average diameter of 3.0 ± 0.58 μm (*n* = 18).

### Projection patterns of OA-L-IR neurons

The connections of OA-L-IR neurites to their corresponding cell bodies were mostly invisible but some projections into neuropil areas could be distinguished. The most pronounced neurite (Fig. 4j) in our preparations was a projection that appears similar to the umbrella-shaped projection (OA-USP) that has previously been described for *Nasonia* (Haverkamp and Smid 2014). This neurite originates at the ventro-medial side of the brain, although a connection with cell bodies was not visible. It passes very close to the posterior side of the OA-3 cluster and then projects in a dorsal direction along the brain midline, close to the oesophageal foramen. The neurite bends at the dorso-posterior side of the brain and runs in an ipsilateral direction where it bifurcates (Fig. 4f) and continues in the direction of (but could not be observed to innervate) the mushroom bodies.

Several neurites form a network that surrounds the oesophageal foramen. The origin of these neurites could not be traced but they are located close to clusters OA-3 and OA-VUM and may therefore originate at these clusters. Neurites from this network innervate the antennal lobe, the mushroom bodies and the optic lobes. The most anterior neurite from this network projects laterally and innervates the antennal lobe at its posterior side (Fig. 4j). Another neurite from the anterior side of the network projects dorso-laterally and innervates the mushroom body calyx. This neurite resembles the stag-like projection (OA-SLP) that was described for *Nasonia* (Haverkamp and Smid 2014). It is less pronounced than OA-USP and too faint to follow in z-stack projections.

The optic lobes are innervated by a neurite that projects from the ventro-anterior to the dorso-posterior side of the oesophageal foramen (Fig. 3f). Close to the posterior cuticle, the neurite bends and projects laterally towards the optic lobes. It bifurcates into a neurite that innervates the dorso-posterior side of the medulla and into a neurite that follows the medial rim of the lobula in an anterior direction and innervates the ventro-anterior side of the medulla. Two distinct layers of sparsely distributed varicose terminals are visible in the medulla (Fig. 4g).

Little variation could be detected in the density of the OA-L-IR varicose terminals across the various neuropil areas. The overall density of these terminals was lower than the density of the 5HT-L-IR varicose terminals but not a single neuropil area was completely devoid of OA-like immunoreactivity. In the antennal lobes, the density of varicose terminals was higher in the centre than at the rim. Specific innervation of antennal lobe substructures, such as glomeruli, could not be analysed in these preparations.

The central complex shows pronounced varicose terminals, especially in the ellipsoid body (Fig. 4f). This high density of varicose terminals makes the ellipsoid body stand out from the surrounding tissue. An arch of varicose terminals surrounds the central complex dorsal to the fan-shaped body (Fig. 4f). The protocerebral bridge is also clearly visible because of the high density of the varicose terminals (Fig. 4h). Several varicose terminals lie in the centre and rim of the mushroom body calyx (Fig. 4i), whereas the mushroom body lobes cannot be distinguished from the surrounding neuropil tissue because of the similarities in the intensity of the background staining.

### Distribution of DA-L-IR neurons

**Fig. 5:**
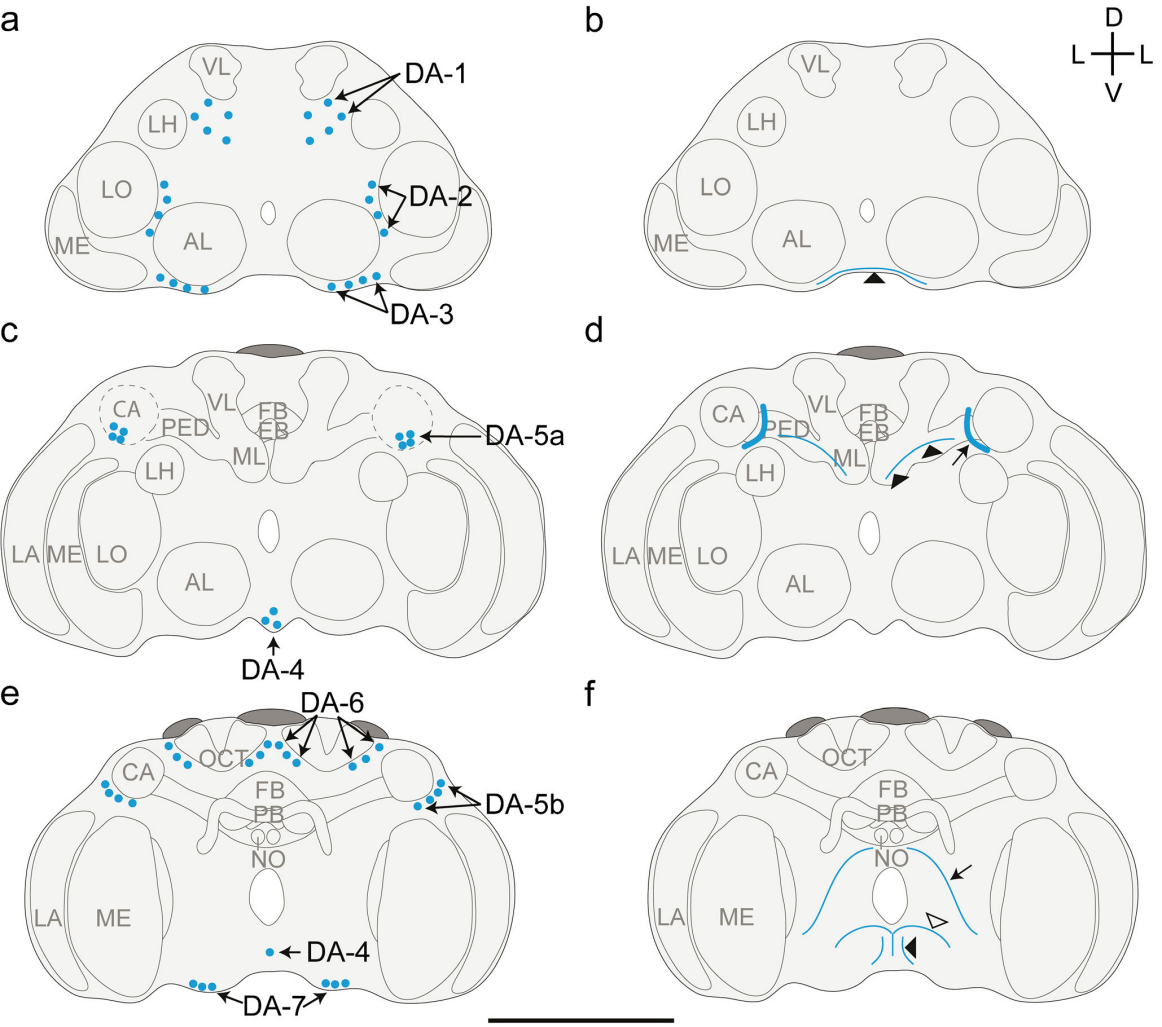
Representation of the location of dopamine-like immunoreactive (DA-L-IR) cell bodies (**a**, **c**, **e**) and projections (**b**, **d**, **f**) in the brain of *T. evanescens*, shown dorsal side up at three locations through the depth of the brain. **a**, **b** Anterior view at approximately one quarter through the depth of the brain. **a** Location of clusters DA-1, DA-2 and DA-3. **b** A small network of neurites (*arrowhead*) projects in a medial direction along the ventral rim of the brain. **c**, **d** Anterior view halfway through the depth of the brain. **c** Location of clusters DA-4 and DA-5a. Cluster DA-5a is located anterior to the calyx (*CA*, *dashed outline*). **d** A thick bundle of DA-L-IR fibres (*arrow*) runs anterior to the mushroom body pedunculus and medial to the calyx. A single neurite (*arrowheads*) projects along the pedunculus in the direction of the calyx. **e**, **f** Posterior view three quarters through the depth of the brain. **e** Location of clusters DA-4, DA-5b, DA-6 and DA-7. **f** A DA-L-IR neurite (*arrow*) projects ventro-laterally from the dorsal side of the oesophageal foramen to the ventral side of the oesophageal foramen. A single neurite (*open arrowhead*) projects dorsally along the brain midline and bifurcates just ventral to the oesophageal foramen. The most posterior neurite (*black arrowhead*) projects dorsally from the ventral rim of the brain and may innervate the thoracic ganglia (*AL* antennal lobe, *LA* lamina, *ME* medulla, *LO* lobula, *LH* lateral horn, *PED* pedunculus, *VL* vertical lobe, *ML* medial lobe, *FB* fan-shaped body, *EB* ellipsoid body, *PB* protocerebral bridge, *NO* noduli, *OCT* ocellar tract, *D* dorsal, *V* ventral, *L* lateral). *Bar* 50 μm (for an average-sized brain)

The DA-L-IR staining was less intense than the 5HT-L-IR staining. Many DA-L-IR neuron clusters were visible but only a few neurites (Fig. 5). The average diameter of DA-L-IR cell bodies was 2.3 ± 0.38 μm (*n* = 160). Neurites and varicose terminals were approximately 0.5 μm in diameter. The orientation of dopaminergic neurons in *T. evanescens* differs from the descriptions in *A. mellifera* (Schafer and Rehder 1989; Schürmann et al. 1989) and *D. melanogaster* (Nässel and Elekes 1992; Monastirioti 1999; Mao and Davis 2009). Our numbering of cell clusters, therefore, does not correspond to the numbering that was used for those species.

**Fig. 6:**
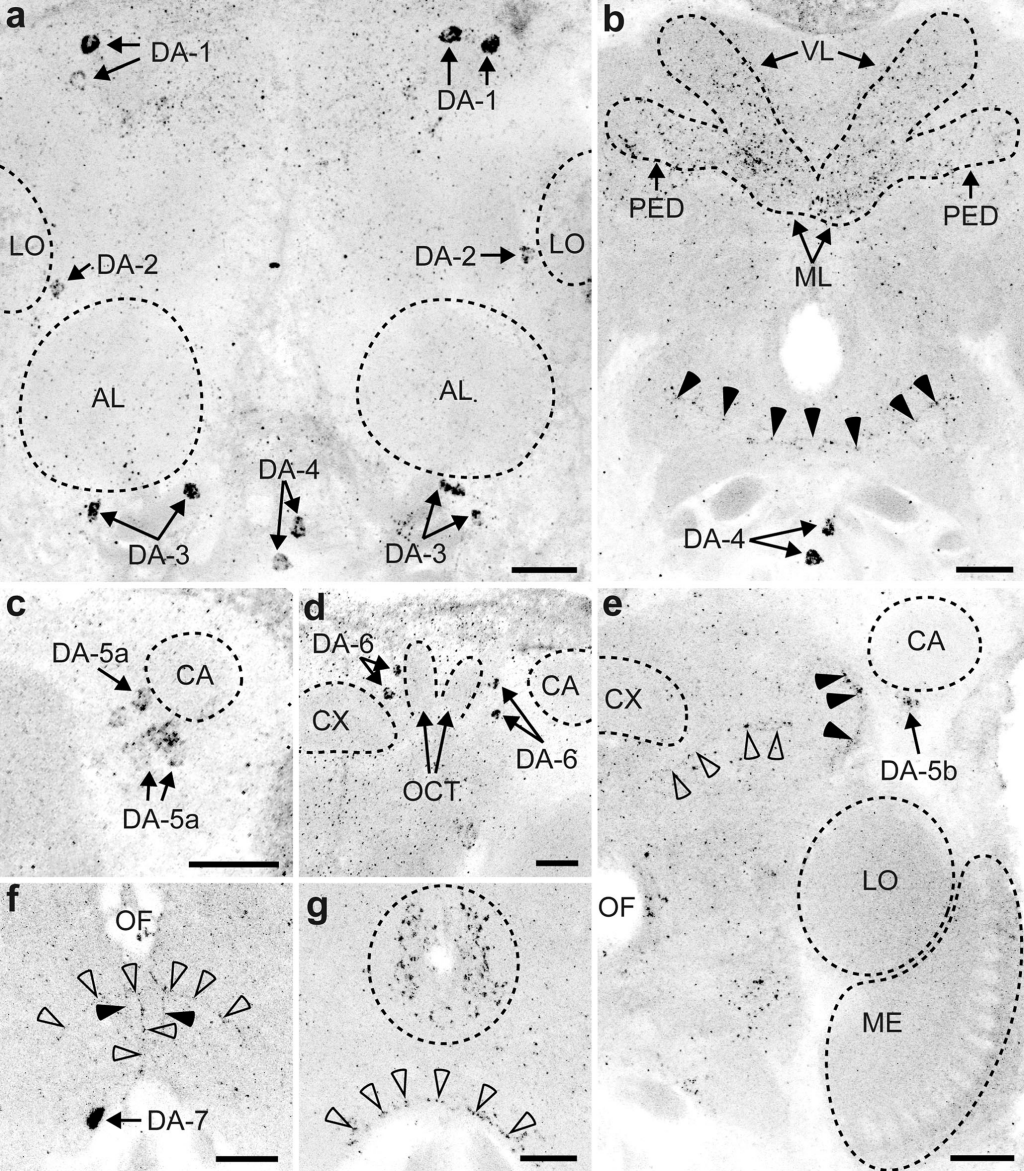
Z-stack projections of confocal images showing dopamine-like immunoreactive (DA-L-IR) cell bodies and projection patterns in *T. evanescens*. **a** Cluster DA-1 (up to five neuron pairs, *arrows*) is located in the latero-anterior central brain. Cluster DA-2 (up to four neuron pairs, *arrows*) is located medial to the lobula (*LO*) and dorso-laterally from the antennal lobe (*AL*). Cluster DA-3 (up to four neuron pairs, *arrows*) is located ventral to the AL. Cluster DA-4 (up to four unpaired neurons, *arrows*) is located medial in the ventral rim of the brain. **b** The pedunculus (*PED*), medial lobes (*ML*) and vertical lobes (*VL*) of the mushroom bodies exhibit higher densities of varicose terminals than the surrounding neuropil. *Arrowheads* indicate a small anterior network of neurites that project in a medial direction, dorsal to DA-4 (*arrows*). **c** Cluster DA-5 consists of up to eight neuron pairs (*arrows*), of which subcluster DA-5a is located on the ventro-anterior side of the calyx (*CA*) and dorsal to the medulla (*ME*). **d** Cluster DA-6 (up to six neuron pairs, *arrows*) are located on the medial side of the CA and surround the ocellar tract (*OCT*). **e** The calyx and optic lobes are the only neuropils that are devoid of DA-like immunoreactivity. Subcluster DA-5b (*arrow*) is located on the ventro-lateral side of the calyx (*black arrowhead* location of the neurite bundle that occurs on the ventro-medial side of the calyx, *open arrowheads* neurite that follows the pedunculus in the direction of the calyx). **f** Cluster DA-7 consists of up to three neuron pairs (*arrow*) located posteriorly in the ventral rim of the brain. The most posterior neurite runs parallel to the brain midline from the ventral rim of the brain in the direction of the oesophageal foramen (*black arrowheads*). Medial to this neurite, another neurite (*open arrowheads*) follows the brain midline and bifurcates just ventral to the oesophageal foramen. **g** Varicose terminals surround the oesophageal foramen (*outlined area*) and are present in the ventral rim (*arrowheads*) of the brain (*CX* central complex). Figures are oriented with the dorsal side pointing upwards. Contrast of z-stack projections was enhanced in Adobe Photoshop CS6. *Bars* 10 μm

Cluster DA-1 (Fig. 6a) is the most anterior cell cluster, located latero-anteriorly in the central brain, directly underneath the frontal cuticle. We counted up to 5 pairs of neurons in this cluster. On average, 2.6 ± 0.89 (*n* = 27) neurons were present, with an average diameter of 2.5 ± 0.33 μm (*n* = 27). Cell bodies of this cluster are somewhat scattered, being located 3–16 μm apart from each other.

Cluster DA-2 (Fig. 6a) lies posterior to cluster DA-1, medial to the lobula and dorso-lateral to the antennal lobes. It consists of up to four pairs of neurons and, on average, 2.3 ± 0.86 (*n* = 28). Their average diameter was 2.3 ± 0.35 μm (*n* = 28).

Cluster DA-3 (Fig. 6a) is located in the ventral rim of the brain, ventral to the antennal lobes. We counted up to four pairs of neurons in this cluster and, on average, 2.7 ± 0.66 (*n* = 29). Their diameter was 2.0 ± 0.24 μm (*n* = 29).

Cluster DA-4 (Fig. 6a) is located medially in the ventral rim of the brain, at an approximately similar location as OA-VUM. This cluster consists of up to four unpaired neurons. Sometimes, one of these unpaired neurons is located more posteriorly and we consider this part of the same cluster, because this neuron is unpaired and occurs in the same ventro-medial location. On average, 2.5 ± 0.82 (*n* = 25) neurons were present, with an average diameter of 2.5 ± 0.49 μm (*n* = 25).

Cluster DA-5 is located ventral and posterior to the lateral rim of the calyx and dorsal to the lobula. It consists of up to eight pairs of neurons and, on average, 3.9 ± 2.16 (*n* = 28). The average diameter of these neurons was 2.2 ± 0.33 μm (*n* = 28). This cluster appears to consist of two subclusters, indicated as DA-5a (Fig. 6c) and DA-5b (Fig. 6e). Cell bodies of DA-5a are oriented in a cluster ventro-anterior to the calyx. Slightly more posteriorly, cell bodies of cluster DA-5b are oriented in a dorso-ventral line at the lateral rim of the calyx. The two subclusters are located very close together and we therefore do not distinguish between them in our analyses.

Cluster DA-6 (Fig. 6d) is located posterior to the calyx and the central complex. Cell bodies of this cluster are positioned on the medial and lateral sides of the ocellar tract. We counted up to six pairs of neurons in this cluster (on average 3.2 ± 1.74; *n* = 13), with an average diameter of 2.3 ± 0.32 μm (*n* = 13).

Cluster DA-7 (Fig. 6f) is the most posterior dopaminergic cell cluster, being located in the ventro-posterior rim of the brain. This cluster contained up to three pairs of neurons (on average 1.4 ± 0.70; *n* = 10). The average diameter of these neurons was 2.4 ± 0.26 μm (*n* = 10).

### Projection patterns of DA-L-IR neurons

Projections of DA-L-IR neurons were sparsely visible. The connections of the neurites to the corresponding cell bodies could not be traced in any of the clusters. The most pronounced DA-like immunoreactivity was found at the ventral base of the mushroom body calyx, where a bundle of DA-L-IR fibres (approximately 1.4 μm in diameter) was located anterior to the mushroom body pedunculus and medial to the calyx (Fig. 6e). It was closely located to cluster DA-5 but we could not observe a connection. Close to this bundle, a single neurite appeared to project dorso-laterally in the direction of the calyx (Fig. 5d).

On the anterior side of the brain, we found a small network of neurites that projected in a medial direction through the ventral rim of the brain (Fig. 6b). These neurites were located ventro-posteriorly to the antennal lobes and medially to the neurons of the DA-3 cluster. Although these neurites might originate from the DA-3 neurons, this could not be observed.

The most posterior neurite runs parallel to the brain midline, from the ventral rim of the brain in the direction of the oesophageal foramen (Fig. 6f). This neurite may innervate the thoracic ganglia. Medial to this neurite, another neurite follows the brain midline and bifurcates just ventrally to the oesophageal foramen (Fig. 6f). The bifurcations bend and project in a ventro-lateral direction, where they could not be traced further. Another neurite projects ventro-laterally from the dorsal side of the oesophageal foramen (ventro-posterior to the medial mushroom body lobe) to the ventral side of the oesophageal foramen (Fig. 5f). This neurite could not be traced further. Neurites innervating other major neuropil areas (i.e., optic lobes, antennal lobes, lateral horn and central complex) were not visible.

The density of DA-L-IR varicose terminals was lower than the densities of 5HT- and OA-L-IR terminals. The entire brain appeared equally innervated by similar low levels of varicose terminals. Only the mushroom body calyces and optic lobes seemed to be completely devoid of varicose terminals (Fig. 6e). Higher densities of varicose terminals were visible in the pedunculus, in the medial and vertical lobes of mushroom bodies (Fig. 6b) and in the ventro-posterior part of the brain (in the ventral rim of the brain and surrounding the oesophageal foramen; Fig. 6g).

## Discussion

Our study provides the first description of the morphology of 5HT-, OA-, and DA-L-IR neurons in the brains of the minute parasitic wasp *T. evanescens*. In the sections below, we show that these miniaturized brains contain comparable numbers of monoaminergic neurons to those of much larger insects, namely *A. mellifera*, *D. melanogaster* and larger parasitic wasps of the genera *Nasonia* and *Cotesia*. Some neuron clusters in *T. evanescens* contain similar numbers of neurons as comparable clusters in larger insects, whereas others contain fewer neurons and yet others are entirely absent. The 5HT-L-IR neuron clusters appear to be especially conserved in complexity. Some 5HT-L-IR clusters that are present in other insects are absent in *T. evanescens* but most of the remaining clusters contain a similar number of neurons as in other species. Additional differences have been observed between the OA-L-IR neuron clusters of *T. evanescens* and those of the larger insects, although the distribution and number of OA-L-IR neurons is very similar to descriptions of the related parasitic wasps of the genus *Nasonia*. The complexity of DA-L-IR neuron clusters appears to be severely reduced compared with that of other insects. We will elaborate on the differences in the distribution, number and size of the neurons between *T. evanescens* and other insects in the following sections.

### Immunohistochemistry

The OA-L-IR staining was less intense in *T. evanescens* than in a previous study of the larger parasitic wasps of the genus *Nasonia* (Haverkamp and Smid 2014), despite large similarities in the methodologies of these two studies. The lower intensity in *T. evanescens* may be related to the small size of neuronal cell bodies and neurites in this wasp and to the thin optical sections that were required accurately to visualize them. This indicates that studying the smallest neurons in miniaturized species such as *T. evanescens* is technically challenging.

The 5HT-L-IR staining was more intense than the OA- and DA-L-IR staining in our study. More 5HT is possibly present in the wasp brains than OA and DA, although the titres of DA are much higher than the titres of OA and 5HT in the brains of honeybees, bumblebees and ants (Harris and Woodring 1992; Bloch et al. 2000; Cuvillier-Hot and Lenoir 2006). Methodological differences might provide alternative explanations for the higher detectability of 5HT than of OA and DA. The methods to visualize OA and DA involved the use of a glutaraldehyde-based fixative, which crosslinks proteins more strongly than the formaldehyde-based fixative used for 5HT-like immunoreactivity (Hopwood 1967). Strong crosslinking might have reduced the permeability of the tissues and partially masked antigens in a more severe way than occurred during the procedures to visualize 5HT. Furthermore, antibodies against OA and DA do not bind the oxidized form of their target amines, whereas this problem does not arise for antibodies against 5HT. Although we used sodium borohydride to reduce the oxidized forms, the efficacy of this method is not clear.

Antibodies against enzymes that are involved in the biosynthesis of OA and DA may provide complementary data to aid the identification of OA- and DA-L-IR neurons. Antibodies against tyramine beta-hydroxylase have been used successfully to reveal OA-like immunoreactivity (Monastirioti et al. 1996; Koon et al. 2011; Wu et al. 2013), whereas antibodies against tyrosine hydroxylase have been used for DA-like immunoreactivity (Nässel and Elekes 1992; Mao and Davis 2009). Use of these antibodies may enhance the detection of OA and DA in future studies in *T. evanescens*.

### Distribution and projections of 5HT-L-IR neurons

We counted up to 38 5HT-L-IR neuron pairs in *T. evanescens* (Table 1). This is comparable with the number of 5HT-L-IR neuron pairs in *D. melanogaster*, in which up to 41 neuron pairs have been counted (Sitaraman et al. 2008). More neuron pairs have been observed in *A. mellifera*: approximately 75 (Schürmann and Klemm 1984). The difference in number of 5HT-L-IR neurons between *T. evanescens* and *A. mellifera* is partially caused by clusters 5HT-4 and 5HT-5. These are not visible in *T. evanescens* but contain up to 24 neuron pairs in *A. mellifera*. Cluster 5HT-1 explains the remaining difference; this cluster contains up to 30 neuron pairs in *A. mellifera* but only up to six pairs in *T. evanescens*. In *D. melanogaster*, this cluster contains up to twice as many neurons as in *T. evanescens*. All other clusters contain an approximately equal number of neuron pairs in *T. evanescens*, *A. mellifera* and *D. melanogaster*. These striking similarities in neuron numbers indicate that the 5HT-L-IR neuron clusters in *T. evanescens* are highly conserved compared with the other insects.

**Table 1:** Comparison of monoaminergic neurons between *Trichogramma evanescens* and larger insects. Total number and diameter of cell bodies immunoreactive for serotonin (*5HT*), octopamine (*OA*) and dopamine (*DA*). Diameters are shown as average values (± SD) or as reported total range

Immunoreactivitity	Number and diameter of cell bodies	*Trichogramma evanescens* (this study)	*Apis mellifera*	*Drosophila melanogaster*	*Nasonia vitripennis* and *Nasonia giraulti*
5HT	Number	38 pairs	75 pairs ^a^	41 pairs ^e^	-
	Diameter	2.1 ± 0.4 μm	8–30 μm ^a^	-	-
OA	Number	16 pairs, 13 unpaired	80 pairs ^b^, 14 unpaired ^c^	41 pairs, 26 unpaired ^f^	24 pairs, 12–14 unpaired ^i^
	Diameter	3.3 ± 0.8 μm	8–45 μm ^b^	5–10 μm ^f^	6–11 μm ^i^
DA	Number	30 pairs, 4 unpaired	119 pairs ^d^	282 pairs ^g^, 2 unpaired ^h^	-
	Diameter	2.3 ± 0.4 μm	8–30 μm ^d^	-	-

Data from: ^a^ Schürmann and Klemm 1984; ^b^ Sinakevitch et al. 2005; ^c^ Schroter et al. 2007; ^d^ Schürmann et al. 1989; ^e^ Sitaraman et al. 2008; ^f^ Sinakevitch and Strausfeld 2006; ^g^ Mao and Davis 2009; ^h^ Budnik and White 1988; ^i^ Haverkamp and Smid 2014

The distribution pattern of 5HT-L-IR neuron clusters in *T. evanescens* largely corresponds to the pattern in *A. mellifera* and *D. melanogaster* (Schürmann and Klemm 1984; Monastirioti 1999; Blenau and Thamm 2011) but some differences were apparent, as described in detail in the Electronic Supplementary Material.

### Distribution and projections of OA-L-IR neurons

We counted up to 16 OA-L-IR neuron pairs and up to 13 unpaired OA-L-IR neurons in the brain of *T. evanescens*. The number of paired OA-L-IR neurons is larger in other insects (Table 1). On average, 24 OA-L-IR neuron pairs are present in the *Nasonia* brain (Haverkamp and Smid 2014), 41 in *D. melanogaster* (Sinakevitch and Strausfeld 2006) and up to 80 in *A. mellifera* (Sinakevitch et al. 2005). This difference in the number of neurons between *T. evanescens* and other insects is partially caused by differences in the number of neurons per cluster and partially by differences in the number of clusters that are present in these species. The clusters observed in both *T. evanescens* and *Nasonia* contain the same numbers of neurons. In contrast, almost all paired clusters in *A. mellifera* and *D. melanogaster* contain more neurons than in *T. evanescens*. However, a remarkable similarity exists in the number of OA-VUM neurons in *T. evanescens* and in other hymenopterans. We counted up to 13 OA-VUM neurons in two well-stained *T. evanescens* brains, a number that is comparable with that in *A. mellifera* (14 neurons; Schroter et al. 2007), *Nasonia* wasps (12–14 neurons; Haverkamp and Smid 2014) and *Cotesia* wasps (14–20 neurons; Bleeker et al. 2006).

The distribution pattern of OA-L-IR neuron clusters in *T. evanescens* largely corresponds to previous findings in the parasitic wasps *N. vitripennis* and *N. giraulti* (Haverkamp and Smid 2014). These similarities can be explained by the close relationship of these parasitic wasps; they both belong to the superfamily Chalcidoidea. The distribution of OA-L-IR neuron clusters in *T. evanescens* is also very similar to the distribution in *A. mellifera* (Kreissl et al. 1994; Sinakevitch et al. 2005) and *D. melanogaster* (Sinakevitch and Strausfeld 2006; Busch et al. 2009). The same clusters are mostly present in the three species but they occur at slightly different locations, namely in more subclusters and with more neurons per cluster in *A. mellifera* and *D. melanogaster*. A full comparison of the distribution of OA-L-IR neurons between *T. evanescens* and other insects can be found in the Electronic Supplementary Material.

### Distribution and projections of DA-L-IR neurons

The most striking difference in DA-like immunoreactivity between *T. evanescens* and other insects is the difference in the total number of DA-L-IR neurons. We counted up to 30 paired and four unpaired DA-L-IR neuron pairs in *T. evanescens*, whereas much higher numbers have been observed in other insects (Table 1). *Apis mellifera* has up to 119 DA-L-IR neuron pairs (Schürmann et al. 1989) and *Calliphora erythrocephala* and *Phormia terraenovae* blowflies up to 152 DA-L-IR neuron pairs (Nässel and Elekes 1992). An antibody against tyrosine hydroxylase, a precursor of dopamine, has revealed 282 immunoreactive neuron pairs in the protocerebrum of *D. melanogaster* (Mao and Davis 2009). Most DA-L-IR neurons have been observed in the locust *Schistocerca gregaria*, which has up to 127 neurons in the midbrain and more than 3000 in the optic lobes (Wendt and Homberg 1992).

We expected that the distribution of DA-L-IR neuron clusters in *T. evanescens* would be similar to the distribution of DA-L-IR clusters in other insects, especially those of other hymenopterans. However, the distribution of dopaminergic neurons in *T. evanescens* differs greatly from the distribution in *A. mellifera* (Schafer and Rehder 1989; Schürmann et al. 1989), *C. erythrocephala* and *P. terraenovae* blowflies (Nässel and Elekes 1992), *D. melanogaster* (Budnik and White 1988; Monastirioti 1999; Mao and Davis 2009) and locusts (Wendt and Homberg 1992). The comparison with other insects is further complicated by the lack of connections of DA-L-IR neurites to cell bodies in *T. evanescens*. This obstructs the identification of the similarities in neuron clusters across insects based on similarities in the areas that they innervate. A different antibody, for example, against tyrosine hydroxylase, might reveal more DA-like immunoreactivity and aid the comparison with other species. A full comparison of the distribution of DA-L-IR neurons between *T. evanescens* and other insects can be found in the Electronic Supplementary Material.

### Neuron numbers in comparison with those of other insects

Overall, our study shows that miniaturized *T. evanescens* brains contain comparable numbers of monoaminergic neurons to those of much larger insects. This is surprising, given the difference in the total number of neurons between *T. evanescens* and larger insects. For example, the total number of neurons in the brains of *A. mellifera* has been estimated to be around 960,000 (Menzel and Giurfa 2001). This is approximately 26 times more than the 37,000 neurons that have been estimated to be present in the brains of *T. evanescens* (Makarova and Polilov 2013). However, when comparing the number of monoaminergic neurons of *T. evanescens* with those of *A. mellifera*, much smaller differences are found. *Apis mellifera* have only approximately twice as many 5HT-L-IR neurons (Schürmann and Klemm 1984), 3.5 times as many DA-L-IR neurons (Schürmann et al. 1989) and five times as many OA-L-IR neurons (Sinakevitch et al. 2005). This indicates that a certain level of neural complexity is required to preserve the performance of the monoaminergic neurons. The maintenance of such a high level of complexity may have been enabled by more extreme reductions in the numbers of other types of neurons and by the miniaturization of neuron size (on which we elaborate in the section below).

Of the three types of monoaminergic neurons that we have studied, the 5HT-L-IR neuron clusters appear to be the most conserved. The comparison of 5HT-L-IR neuron counts shows a striking similarity in the number of neurons in all clusters that are present in *T. evanescens* and other insects, except for 5HT-1. This conserved distribution of 5HT-L-IR neurons indicates that modifications to these clusters would compromise vital physiological functions. Differences in the total cell count of 5HT-L-IR neurons between *T. evanescens* and other insects are mostly caused by clusters that are absent in *T. evanescens* and present in other insects and by the difference in neuron numbers of the cluster that innervates the optic lobes (5HT-1). In *A. mellifera*, this cluster contains approximately five-fold more neuron pairs than in *T. evanescens* and, in *D. melanogaster*, this cluster contains approximately two-fold more neuron pairs than in *T. evanescens*. The optic lobes have a strong columnar structure, which relates to the organization of the ommatidia in compound eyes (Paulk et al. 2013). The number of 5HT-L-IR neurons that modulate the functioning of the optic lobes may be directly related to the size of the eye and to the number of ommatidia. Hence, the differences in the numbers of ommatidia between *T. evanescens* (approximately 128; Fischer et al. 2011), *D. melanogaster* (approximately 750; Paulk et al. 2013) and *A. mellifera* (approximately 4500; Srinivasan 2010) may underlie the differences in numbers of 5HT-1 neurons between these insects.

The OA-L-IR neuron clusters appear to be less conserved than the 5HT-L-IR clusters, although large similarities exist in the number of neurons in those OA-L-IR neuron clusters that are present in both *T. evanescens* and the related parasitic wasp *Nasonia*. Only two clusters that are visible in *Nasonia* are absent in *T. evanescens*: OA-0 and OA-4. Almost all paired OA-L-IR clusters in *A. mellifera* and *D. melanogaster* contain more neurons than in *T. evanescens* and *Nasonia*, except for clusters OA-3 and OA-VUM. Cluster OA-3 is the only paired OA-L-IR neuron cluster that consists of an approximately equal number of neurons in *T. evanescens*, *Nasonia* (Haverkamp and Smid 2014), *Cotesia* (Bleeker et al. 2006), *A. mellifera* (Sinakevitch et al. 2005) and *D. melanogaster* (Busch et al. 2009). The number of OA-VUM neurons in *T. evanescens* is similar to the number of OA-VUM neurons described in other hymenopterans, i.e., *A. mellifera* (Schroter et al. 2007), *Nasonia* (Haverkamp and Smid 2014) and *Cotesia* (Bleeker et al. 2006).

The great similarity in numbers of neurons in OA-3 and OA-VUM in *T. evanescens* and other insects indicates that adequate functioning requires a conserved number of neurons, despite large differences in brain size. Neurites of cluster OA-3 and OA-VUM might contribute to the network of neurites around the oesophageal foramen and may have vital functions for the neuropil areas that they innervate (i.e., optic lobes, mushroom bodies and antennal lobes). Furthermore, OA-VUM neurons are important in the neural processing pathways that lead to memory formation in insects (Hammer and Menzel 1995; Schroter et al. 2007). The conservation of OA-VUM neuron numbers among hymenopterans has been hypothesized to be related to the complex learning abilities that are required for a parasitic life style (Haverkamp and Smid 2014), which evolved at the base of the Euhymenoptera (Whitfield 2003; Farris and Schulmeister 2011). The conserved number of OA-VUM neurons in bees and wasps, including the miniaturized *T. evanescens*, which has a brain volume that is approximately 2500× smaller than that of *A. mellifera* (Mares et al. 2005; Van der Woude et al. 2013), supports this hypothesis.

The DA-L-IR neuron clusters appear to be the least conserved of the three monoaminergic systems that we have studied. A large difference in the distribution of DA-L-IR neurons exists between *T. evanescens* and other insects and, therefore, most clusters cannot be directly compared. Furthermore, the total number of DA-L-IR neurons is much higher in other insects. This indicates that a severe modification of the dopaminergic neuron clusters facilitated the evolution of small brain sizes and that these modifications were possible without compromising vital physiological functions.

### Neuron size in comparison with other insects

As expected, monoaminergic neurons are smaller in *T. evanescens* than in larger insects. For example, the diameter of OA-L-IR cell bodies is on average 3.3 μm in *T. evanescens*, 6–11 μm in *Nasonia* wasps (Haverkamp and Smid 2014), 5–10 μm in *Cotesia* wasps (Bleeker et al. 2006), 5–10 μm in *D. melanogaster* (Sinakevitch and Strausfeld 2006) and 8–45 μm in *A. mellifera* (Sinakevitch et al. 2005). The diameters of DA- and 5HT-L-IR cell bodies are even smaller: on average 2.3 μm and 2.1 μm, respectively, in *T. evanescens* and 8–30 μm in *A. mellifera* (Schürmann and Klemm 1984; Schafer and Rehder 1989; Schürmann et al. 1989). More accurate comparisons of neuron size between species will require volumetric data on cell body and brain volumes. Such comparisons might reveal whether neuronal cell bodies are miniaturized to a greater or lesser extent than would be expected from the differences in brain size between *T. evanescens* and larger insects.

Neuronal cell bodies have previously been reported to range between 1.2 and 2.8 μm in diameter in *T. evanescens* (Makarova and Polilov 2013). The monoaminergic cell bodies that we measured in our study are larger, ranging in diameter between 1.4 and 5.7 μm (Van der Woude and Smid 2017). This indicates that monoaminergic cell bodies are larger than those of other types of neurons. However, cell body diameters in our study may also be larger because we included wasps of up to 0.9 mm in body length.

Neuronal cell body diameters in *T. evanescens* are among the smallest that have been described in insects, a finding that may be a consequence of brain miniaturization (Niven and Farris 2012; Makarova and Polilov 2013). The smallest insects show a strongly reduced volume of cytoplasm in their neurons (Makarova and Polilov 2013). As a result, the nucleus can occupy up to 90% of the volume of the neuronal cell body (Polilov 2005). This indicates that the size of the nucleus limits neuronal cell body size. The volume of the nucleus in turn is related to the size of the genome (Gregory 2001). Genome size has, to our knowledge, not been established for *T. evanescens*. However, genome sizes of related species are surprisingly similar to those of larger insects. For example, *Trichogramma platneri* has a similar genome size (i.e., ∼176 Mb; Ardila-Garcia et al. 2010) to that of *D. melanogaster* (i.e., ∼180 Mb; Adams et al. 2000) and the genome of *Trichogramma brassicae* has been found to be similar in size (i.e., ∼246 Mb; Johnston et al. 2004) to the genome of *A. mellifera* (i.e., ∼235 Mb; Ardila-Garcia et al. 2010). Hence, the smaller size of cell bodies in *T. evanescens* compared with those of *A. mellifera* and *D. melanogaster* (as outlined above) may not be caused by a difference in genome size. The evolutionary process of miniaturization of neuron size may instead have resulted in densely packed chromatin inside the nucleus (Makarova and Polilov 2013; Polilov 2015). Further miniaturization of cell body size may require modifications that also negatively affect the functionality of the neurons, such as the lysis of neuronal nuclei (Polilov 2012). Lower numbers of neurons or neuron clusters, such as we observed in this study, may have been a necessary modification that prevented the loss of functionality of neurons during the evolutionary process of brain miniaturization in *T. evanescens*.

The average diameter of OA-L-IR cell bodies is considerably larger than that of 5HT- and DA-L-IR cell bodies: approximately 53% and 42% larger, respectively. A similar trend has been noted in *A. mellifera*: OA-L-IR cell bodies can reach diameters of up to 45 μm (Sinakevitch et al. 2005), whereas the largest 5HT- and DA-L-IR cell bodies have a diameter of 30 μm (Schürmann and Klemm 1984; Schafer and Rehder 1989). This difference in neuron size is not reflected by the size of the varicose terminals. The 5HT-L-IR varicose terminals are almost twice as large (approximately 1 μm in diameter) as the OA- and DA-L-IR terminals (0.6 and 0.5 μm, respectively), indicating that the release sites of 5HT-L-IR neurons are larger.

### Concluding remarks

Our study shows that the monoaminergic neuron systems in the minute brain of *T. evanescens* are highly comparable in complexity to those in much larger insects. However, reductions of complexity do indeed occur in the neuronal systems that we studied, possibly as a consequence of the miniaturized brain sizes in these wasps. Monoaminergic cell body diameters are smaller in *T. evanescens* than in larger insects. Miniaturization of neuron size may have enabled the maximized complexity of neuronal systems; the monoaminergic neuron clusters contain more neurons than expected from the differences in total number of neurons in the brains of *T. evanescens* and larger insects. We observed that these reductions in neuron numbers are not proportional but vary in the different monoaminergic systems.

Some neuron clusters are similar in complexity as those in larger insects, whereas other clusters are partially reduced and yet others are entirely absent in *T. evanescens*. The neuron clusters of which the complexity has been maintained are mostly serotonergic, together with some octopaminergic clusters. The complexity of these clusters may have been maintained because they play key roles in brain performance. The clusters that are partially reduced or completely absent are mostly dopaminergic, together with some octopaminergic clusters. Modifications of these clusters may have facilitated brain miniaturization and appear to have been possible without any compromise to vital brain functions.

The results of our study reveal some of the evolutionary adaptations that may facilitate optimal behavioural and cognitive complexity with respect to miniaturized brains. These results are especially interesting in comparison with the modification of monoamine neuron clusters that arise as a result of intraspecific differences in body size between small and large sister wasps (Van der Woude and Smid 2017). Further research should unravel the functional consequences of the absence of some neuron clusters and innervation patterns in *T. evanescens* in comparison with those of larger insects, such as the unique absence of 5HT-like innervation of the antennal lobe. Furthermore, a comparison of the numbers of 5HT- and DA-L-IR neurons would be of interest between *T. evanescens* and the related but larger *Nasonia* parasitic wasps, which show a great similarity in their OA-L-IR neuron distribution. This might reveal whether the numbers of the different monoaminergic neurons are similarly conserved between these two species.

## Electronic supplementary material

ESM 1 (DOCX 31 kb)
