# Supplementary material for: Maximized complexity in miniaturized brains: morphology and distribution of octopaminergic, dopaminergic and serotonergic neurons in the parasitic wasp, Trichogramma evanescens
Source: Cell Tissue Res. 2017 Jun 9;369(3):477–96. doi: 10.1007/s00441-017-2642-8 (PMC5579201; doi:10.1007/s00441-017-2642-8)
Supplement: Supplementary file 1 — (DOCX 31 kb) [file 441_2017_2642_MOESM1_ESM.docx]

**Maximized complexity in miniaturized brains: morphology and distribution of octopaminergic, dopaminergic and serotonergic neurons in *Trichogramma evanescens* parasitic wasps**

Cell and Tissue Research

Emma van der Woude^1^, Hans M. Smid^1^

^1^ Laboratory of Entomology, Wageningen University, P.O. Box 16, 6700 AA, Wageningen, the Netherlands; emmavanderwoude@gmail.com

**Electronic Supplementary material**

*Distribution and projections of 5HT-L-IR neurons in comparison to other insects*

The distribution pattern of 5HT-L-IR neuron clusters in *T. evanescens* largely corresponds to the pattern in *A. mellifera* and *D. melanogaster* (Schürmann and Klemm, 1984; Monastirioti, 1999; Blenau and Thamm, 2011), but there were some differences. Cluster 5HT-0 in *T. evanescens* has not been described for *A. mellifera* (Schürmann and Klemm, 1984), but the location of this cluster appears similar to the most anterior cell cluster in *D. melanogaster* in the anterior lateral protocerebrum (Blenau and Thamm, 2011). Furthermore, we did not observe the deutocerebral giant cell that was described for *A. mellifera* (Rehder et al., 1987), nor the clusters in the anterior medial and posterior lateral protocerebrum that were described for *D. melanogaster* (Blenau and Thamm, 2011).

The deutocerebral giant cell innervates the antennal lobes in *A. mellifera* (Rehder et al., 1987; Seidel and Bicker, 1996). Interestingly, the absence of this neuron in *T. evanescens* co-occurs with an absence of 5HT-L-IR neurites and varicose terminals in the antennal lobes. The cell body of the neuron that innervates the antennal lobes could also not be found in *Harpegnathos saltator* ants, but serotonergic innervation of the antennal lobe was present in this species (Hoyer et al., 2005) and all other insects species studied so far (Dacks et al., 2006). Serotonergic antennal lobe innervation was found to be sparse and incomplete in various families of parasitic wasps, but never completely absent (Dacks et al., 2006). Our study suggests that chemical modulation of antennal lobe neurons may not be serotonergic in *T. evanescens*.

Cluster 5HT-1 is located anteriorly between the lobula and medulla in *T. evanescens*. This cluster has previously been described in *A. mellifera*, where it occurs in two subclusters: one located directly between the lobula and medulla, and one that is located more ventrally at the rim of the brain (Schürmann and Klemm, 1984). In *D. melanogaster,* a single 5HT-L-IR neuron cluster has been described at a similar location*,* located in the lateral protocerebrum between the medulla and central neuropil (Blenau and Thamm, 2011).

The innervation pattern of the optic lobes shows a single layer of varicose terminals in both the medulla and lamina of *T. evanescens,* which corresponds to observations in *A. mellifera* (Schürmann and Klemm, 1984; Nässel, 1988). However, the origin of innervation of the medulla and lamina in *T. evanescens* differs from the descriptions for *A. mellifera* and *D. melanogaster.* In *A. mellifera* and *D. melanogaster*, the optic lobes are only innervated by neurites of the clusters that correspond with 5HT-1 (Schürmann and Klemm, 1984; Nässel, 1988), whereas cluster 5HT-2 also contributes to optic lobe innervation in *T. evanescens*. The neurites of cluster 5HT-2 could not be traced in *A. mellifera* (Schürmann and Klemm, 1984), and a similar cluster has not been described for *D. melanogaster* (Blenau and Thamm, 2011). The involvement of cluster 5HT-2 in the innervation of the optic lobes of *T. evanescens* suggests that this cluster has similar functions as cluster 5HT-1, which has been hypothesized to modulate optic lobe neurons, visual processing and diurnal activity (Nässel et al., 1985; Nässel, 1988).

Cluster 5HT-3 had most cell bodies in *T. evanescens,* and neurites that innervated many neuropil areas by projecting in lateral, dorso-anterior and ventro-anterior direction. A similar cluster is described in *A. mellifera*, which innervates anterior neuropil areas (Schürmann and Klemm, 1984). Cluster 5HT-3 could be similar to the cluster described in the posterior medial protocerebrum in *D. melanogaster*, which is located in the posterior cell body rind, medial to the calyx (Blenau and Thamm, 2011).

The mushroom bodies are among the neuropil areas that are innervated by 5HT-3 neurites. A single neurite enters the mushroom bodies through the pedunculus and bifurcates inside the calyx. The other components of the mushroom bodies lack serotonergic innervation. This is in contrast to the mushroom body innervation pattern that was described for *A. mellifera* (Schürmann and Klemm, 1984). Here, the calyces completely lack 5HT-like immunoreactivity, whereas the pedunculus, medial-, and vertical lobes contain a pronounced pattern of layered 5HT-like immunoreactivity. Similar mushroom body innervation patterns were shown in ants, but here the calyx is innervated by a few neurites (Hoyer et al., 2005). These differences could suggest that modulation of mushroom body functioning differs between hymenopterans.

We did not observe the 5HT-4 and 5HT-5 clusters that were described for *A. mellifera* (Schürmann and Klemm, 1984). Cluster 5HT-4 is located in the pars intercerebralis in *A. mellifera*, at the posterior medial rim of the medial calyx. Cluster 5HT-5 is located more posteriorly in the pars intercerebralis in *A. mellifera*, ventral to 5HT-4. These clusters may be completely lacking in *T. evanescens.* Alternatively, the 5HT-4 and 5HT-5 clusters could be located too close to the cluster of 5HT-3 neurons to distinguish between them, because the second calyx that is present in *A. mellifera* is absent in *T. evanescens*. In *A. mellifera,* the 5HT-4 cluster causes the layered innervation pattern of the mushroom bodies (Schürmann and Klemm, 1984), and innervates the central complex (Seidel and Bicker, 1996). This layered mushroom body innervation was absent in *T. evanescens*. The central complex did show 5HT-L-IR innervation, but the origin could not be traced.

We observed three clusters of 5HT-L-IR cell bodies in the ventral rim of the brain of *T. evanescens*, and grouped these as 5HT-6. This corresponds to findings in *A. mellifera*, where the labial, maxillary and mandibular neuromeres of the suboesophageal zone each contain a cluster of 5HT-L-IR cell bodies (Rehder et al., 1987; Seidel and Bicker, 1996). There are also three clusters at similar locations in *D. melanogaster* (Monastirioti, 1999; Blenau and Thamm, 2011). There was a difference in the number of cell bodies per cluster between *A. mellifera* and *T. evanescens.* In *A. mellifera* there are only two cell body pairs per cluster (Blenau and Thamm, 2011), whereas we counted up to four pairs per cluster in *T. evanescens.* There may be some variability in our neuron counts that is caused by different clusters lying too close together to distinguish between in some of the samples. However, many brains have more than six 5HT-6 neurons in total. This indicates that cluster 5HT-6 does contain more neurons in *T. evanescens* than in *A. mellifera*.

The lateral 5HT-6a neuron projects dorsally towards the brain midline. This projection pattern is in contrast to that of the other 5HT-6 neurons, which project medially and form a network of bifurcations in the ventral rim of the brain, similar as in *A. mellifera* (Rehder et al., 1987). We cannot exclude that the lateral 5HT-6a neuron is part of a different cell cluster. The location of the lateral 5HT-6a neuron resembles the location of the ventral 5HT-1 cluster in *A. mellifera* (Schürmann and Klemm, 1984) and the deutocerebral giant interneuron (Rehder et al., 1987). However, the projection patterns of both clusters differ from the projection that was observed for the lateral 5HT-6a neuron: the ventral 5HT-1 neurons project towards the optic lobes (Schürmann and Klemm, 1984), and the deutocerebral giant interneuron innervates the antennal lobe (Rehder et al., 1987). We decided to consider the lateral 5HT-6a neuron as part of the 5HT-6a cell cluster, because it is located very close to the medial 5HT-6a neuron and cannot be distinguished from it when its neurites are not visible.

*Distribution and projections of OA-L-IR neurons in comparison to other insects*

The distribution pattern of OA-L-IR neuron clusters in *T. evanescens* largely corresponds to previous findings in the parasitic wasps *Nasonia vitripennis* and *Nasonia giraulti* (Haverkamp and Smid, 2014)*.* These similarities could be explained by the close relation of these parasitic wasps; they both belong to the superfamily Chalcidoidea. Only the clusters that were described in *Nasonia* as OA-0 and OA-4 were not found in *T. evanescens.* Cluster OA-0 was found in only a single preparation of *N. giraulti* (n=20) and not at all in *N. vitripennis* (n=24)(Haverkamp and Smid, 2014). This low detection frequency may explain why we did not find this cluster in *T. evanescens.*

We observed more OA-L-IR clusters in *T. evanescens* than were described for the parasitic wasps *Cotesia glomerata* and *Cotesia rubecula* (Bleeker et al., 2006). Only the clusters that correspond to OA-3 and OA-VUM were described for *Cotesia* wasps, and an additional OA-L-IR cluster in the pars intercerebralis that we did not observe in *T. evanescens.* However, the staining intensity in *Cotesia* was low compared to the intensity in *Nasonia* (Haverkamp and Smid, 2014). The low numbers of neurons that were detected in *Cotesia* may therefore not reflect a difference in OA-like immunoreactivity, but instead relate to methodological differences.

The distribution of OA-L-IR neuron clusters in *T. evanescens* is also very similar to the distribution in *A. mellifera* (Kreissl et al., 1994; Sinakevitch et al., 2005) and *D. melanogaster* (Sinakevitch and Strausfeld, 2006; Busch et al., 2009). Mostly the same clusters are present in the three species, but they occur at slightly different locations, in more subclusters and with more neurons per cluster in *A. mellifera* and *D. melanogaster*. There were some OA-L-IR clusters that were present in *D. melanogaster* and *A. mellifera*, but that we did not observe in *T. evanescens.* These were the dorso-medial OA-4 neuron clusters, the cluster between the lobula and calyx, some of the subclusters, and the ventral paired median neuron cluster in *A. mellifera*.

The most anterior OA-L-IR neuron clusters in *T. evanescens* were OA-1 and OA-2. They appear at similar locations as the equivalent clusters that were described for *Nasonia* wasps*,* and consist of a single neuron pair per cluster in both *T. evanescens* and *Nasonia* (Haverkamp and Smid, 2014)*.* Similar clusters were described for *A. mellifera*, but cluster OA-2 occurs at a more ventral location in *A. mellifera* than in *T. evanescens* (Kreissl et al., 1994; Sinakevitch et al., 2005). In *D. melanogaster,* OA-1 occurs at a similar location as in *T. evanescens*, but OA-2 is located more laterally: between the ventro-medial and lateral protocerebrum (Sinakevitch and Strausfeld, 2006). Clusters OA-1 and OA-2 were not observed in *Cotesia* wasps (Bleeker et al., 2006).

The most pronounced OA-L-IR neuron cluster in *T. evanescens* was OA-3, in the area around the oesophageal foramen. This cluster was also the cluster with the most pronounced OA-like immunoreactivity in *Nasonia* (Haverkamp and Smid, 2014) and *Cotesia* wasps (Bleeker et al., 2006). A similar cluster is located around the oesophageal foramen in *A. mellifera* and *D. melanogaster*, and is divided into anterior and posterior subclusters (Sinakevitch et al., 2005; Sinakevitch and Strausfeld, 2006). Cluster OA-3 was the only paired OA-L-IR neuron cluster that consists of an approximately equal number of neurons in *T. evanescens* and in other insects. We counted up to nine neuron pairs in *T. evanescens,* whereas 11 neuron pairs were counted in *Nasonia* (Haverkamp and Smid, 2014) and *A. mellifera* (Sinakevitch et al., 2005), and up to eight in *Cotesia* (Bleeker et al., 2006) and *D. melanogaster* (Busch et al., 2009).

Cluster OA-5 consists of up to three neuron pairs in *T. evanescens*. A similar cluster with a three neuron pairs has been described for *Nasonia* wasps (Haverkamp and Smid, 2014)*,* but not for *Cotesia* (Bleeker et al., 2006). Cluster OA-5 consists of two subclusters in *A. mellifera* (Sinakevitch et al., 2005). Subcluster OA-5a is located at a similar location as OA-5 in *T. evanescens*, and consists of only a single neuron pair. Subcluster OA-5b is located at a more posterior location, but we did not observe an equivalent neuron cluster in *T. evanescens.* Three subclusters of OA-5 have been described for *D. melanogaster,* of which OA-5a resembles the location of OA-5 in *T. evanescens* most (Sinakevitch and Strausfeld, 2006).

The location of cluster OA-6 in *T. evanescens* (latero-posterior to OA-VUM in the ventral rim of the brain) corresponds to the location of the posterior subcluster of OA-6 in *A. mellifera* (Sinakevitch et al., 2005), and a similar cluster was also found in *D. melanogaster* (Sinakevitch and Strausfeld, 2006). Cluster OA-6 has not been described for *Nasonia* wasps. There are two clusters lateral to the OA-VUM neurons in *Nasonia*: the ventral median paired neuron cluster in the anterior suboesophageal zone and the posterior median paired neuron cluster in the posterior suboesophageal zone (Haverkamp and Smid, 2014)*.* However, these are located ventro-medially, close to the midline, whereas OA-6 is located ventro-laterally in *T. evanescens*.

The location of the most posterior OA-L-IR neuron cluster (OA-7) corresponds to the location of the dorsal median paired neuron cluster in the dorso-posterior suboesophageal zone of *Nasonia* wasps (Haverkamp and Smid, 2014)*.* This cluster has not been described for *A. mellifera* (Kreissl et al., 1994; Sinakevitch et al., 2005), nor for *D. melanogaster* (Sinakevitch and Strausfeld, 2006; Busch et al., 2009).

The OA-VUM neurons in *T. evanescens* lie at the ventral rim of the brain, very close to the mouthparts. The OA-VUM cluster has been described for many insects, for instance in *A. mellifera* (Sinakevitch et al., 2005; Schroter et al., 2007), *Nasonia* (Haverkamp and Smid, 2014), *Cotesia* (Bleeker et al., 2006), *D. melanogaster* (Sinakevitch and Strausfeld, 2006; Busch et al., 2009), *Phaenicia sericata* blowflies (Sinakevitch and Strausfeld, 2006), and *Manduca sexta* hawkmoths (Dacks et al., 2005). The OA-VUM cluster is usually divided into subclusters named after the neuromere in which they occur; either the mandibular, maxillary or labial neuromere of the suboesophageal ganglion. We could not distinguish between different subclusters in *T. evanescens,* because the neurons are too close together. The average count of approximately four OA-VUM neurons in *T. evanescens* was rather low because the area around the mouthparts is fragile and was often damaged in our preparations, but we counted up to 13 OA-VUM neurons in two well-stained brains. This is remarkably similar to the number of OA-VUM neurons that are present in other hymenopterans: i.e. 14 in *A. mellifera* (Schroter et al., 2007), 12-14 in *Nasonia* wasps (Haverkamp and Smid, 2014) and 14-20 in *Cotesia* wasps (Bleeker et al., 2006).

The neurites of OA-L-IR neurons were less intensely stained than those of 5HT-L-IR neurons. They could not be traced throughout their entire length, and their connections to cell bodies could not be observed at all. However, most of the neurites that were described for *Nasonia* wasps (Haverkamp and Smid, 2014) were also visible in *T. evanescens.* Only the neurite tracts that project from the OA-VUM in the direction of the oesophageal foramen were not found in *T. evanescens*.

The OA-USP was the most pronounced neurite in *T. evanescens.* Its projection pattern is largely similar to the pattern of OA-USP in *Nasonia,* but differs in one aspect: in *T. evanescens* it does not bifurcate into an ipsilateral and contralateral projecting neurite. Only one hemisphere is innervated by a neurite that bends in ipsilateral direction at the dorsal rim of the brain. In this aspect, the projection pattern of OA-USP in *T. evanescens* seems identical to the projection of OA-VUMmd4 in *A. mellifera* (Schroter et al., 2007). This neurite runs along the brain midline and bends in ipsilateral direction at the dorsal rim of the brain. We did not observe a connection of OA-VUM cell bodies to neurites in *T. evanescens,* and the OA-USP could not be traced further ventrally from the oesophageal foramen. We can therefore only speculate about the equivalence of OA-USP in *T. evanescens* and the OA-VUMmd4 projection in *A. mellifera*.

The network of neurites around the oesophageal foramen was also described for *Nasonia* (Haverkamp and Smid, 2014)*.* Neurites from this network innervate the optic lobes, mushroom bodies and antennal lobes in *T. evanescens.* The neurites that innervate the optic lobes show a generally similar projection pattern in both *Nasonia* and *T. evanescens*, but the projections differ in two points. First, in *T. evanescens* there appears to be only a single neurite that innervates the optic lobes, whereas five neurites are responsible for this in *Nasonia.* Second, only the medulla appears to be innervated in *T. evanescens* by this network, whereas both the lobula and medulla are innervated in *Nasonia.*

The mushroom body calyx of *T. evanescens* is innervated by another neurite of the network around the oesophageal foramen. Its projection pattern resembles the pattern of the stag-like projection (OA-SLP) that was described for *Nasonia* (Haverkamp and Smid, 2014), and the projections from OA-VUMmx1 and OA-VUMmd1 in *A. mellifera* (Schroter et al., 2007). Innervation of the mushroom body calyx by OA-SLP has not been described for *Nasonia*. Instead, this neurite innervates the mushroom body pedunculus. In *A. mellifera*, OA-VUMmx1 and OA-VUMmd1 do innervate the mushroom body calyces. Furthermore, OA-SLP in *Nasonia* and OA-VUMmx1 and OA-VUMmd1 in *A. mellifera* innervate the antennal lobe and lateral horn. We observed a neurite that projects laterally from the network around the oesophageal foramen in *T. evanescens* and innervates the antennal lobe, but we could not distinguish a connection between this neurite and OA-SLP. The neurites that innervate the lateral horn were not visible in *T. evanescens*.

*Distribution and projections of DA-L-IR neurons in comparison to other insects*

The most anterior cell clusters that we observed in *T. evanescens* were not described for *A. mellifera* (Schafer and Rehder, 1989; Schürmann et al., 1989). These are the dorsal DA-1 cluster, and cluster DA-2 medial to the lobula and dorso-lateral to the antennal lobes. These two clusters may be equivalent to the two clusters in the anterior protocerebrum in *D. melanogaster*: the protocerebral anterior medial (PAM) or protocerebral anterior lateral (PAL) cluster (Budnik and White, 1988; Mao and Davis, 2009). The PAM cluster has been shown to be important for both aversive and appetitive learning in *D. melanogaster* (Aso et al., 2010; Burke et al., 2012; Liu et al., 2012; Waddell, 2013). However, cluster DA-2 is located more ventro-laterally than both of these clusters in *D. melanogaster.* Cluster DA-2 may also be equivalent to the protocerebral posterior lateral cluster 2 (PPL2) in *D. melanogaster,* which is located more posteriorly than cluster DA-2 in *T. evanescens,* but resembles DA-2 in its location on the lateral rim of the central brain, medial to the optic lobes (Mao and Davis, 2009).

Cluster DA-3 is located in the ventral rim of the brain, ventral to the antennal lobes. The close location to the antennal lobes suggests that these neurons may be similar to the neurons that were described in the suboesophageal zone of *A. mellifera,* which project in anterior direction and innervate the antennal lobes (Schafer and Rehder, 1989; Schürmann et al., 1989). Although we did not observe any neurites that innervate the antennal lobes in *T. evanescens*, the immunoreactivity that we did observe in the antennal lobes may originate at DA-3. In *D. melanogaster*, two clusters are located ventro-posterior to the antennal lobe. These are the protocerebral posterior medial 3 (PPM3) and protocerebral posterior lateral 3 (PPL3) clusters (Mao and Davis, 2009). The location of these clusters is slightly more posterior than the location of DA-3 in *T. evanescens*, although the more ventral location of the antennal lobe in *T. evanescens* makes it difficult to compare clusters between the two species.

Cluster DA-4 is located medially in the ventral rim of the brain, and consists of up to four unpaired neurons in *T. evanescens*. Three of these neurons are located in the ventro-anterior rim of the brain and another unpaired neuron occurs more posteriorly. A similar situation occurs in *D. melanogaster* and blowflies (Budnik and White, 1988; Nässel and Elekes, 1992). Here, two DA-L-IR neurons are located right next to a ventral unpaired median neuron in the anterior part of the suboesophageal zone, and a second ventral unpaired median neuron is located at a more posterior location. No clusters of ventral unpaired median neurons were described in *A. mellifera* (Schafer and Rehder, 1989; Schürmann et al., 1989).

Cluster DA-5 consists of two adjacent subclusters that are located ventro-anterior and lateral to the calyx and dorsal to the optic lobes. Clusters of DA-L-IR neurons occur at similar locations on the lateral side of the calyces in *A. mellifera* and *D. melanogaster* (Schafer and Rehder, 1989; Schürmann et al., 1989; Mao and Davis, 2009). In *A. mellifera*, cluster C3 is located ventral to the lateral calyx (Schafer and Rehder, 1989), and an additional DA-L-IR cluster was observed lateral to the medial calyx (Schürmann et al., 1989). In *D. melanogaster,* the cluster on the lateral side of calyx is indicated as protocerebral posterior lateral 1 (PPL1) (Mao and Davis, 2009)*.* The PPL1 cluster functions together with PAM in the regulation of appetitive and aversive learning in flies (Aso et al., 2010; Burke et al., 2012; Liu et al., 2012; Waddell, 2013). The DA-5 cluster may perform similar functions in *T. evanescens.*

Cluster DA-6 is located in the neural tissue that surrounds the ocellar tracts, dorso-posterior to the mushroom bodies and the central complex. This cluster could be similar to the DA-L-IR neuron cluster that is located dorso-posterior to the central complex in *A. mellifera* (Schafer and Rehder, 1989; Schürmann et al., 1989), and to two protocerebral posterior medial clusters (PPM 1 and 2) in *D. melanogaster* (Mao and Davis, 2009).

Cluster DA-7 is the most posterior dopaminergic cell cluster, located in the most posterior part of the ventral rim of the brain. This cluster could be similar to the posterior DA-IR-L neuron clusters in the suboesophageal zone of *A. mellifera* (Schafer and Rehder, 1989; Schürmann et al., 1989). Most of these clusters in *A. mellifera* project anteriorly towards the antennal lobes, although the most posterior cluster (that was described as S7 by Schafer and Rehder (1989)) projects laterally and towards the thoracic ganglia. We observed similar lateral projections in the ventral rim of the brain and two neurites that appear to innervate the thoracic ganglia in *T. evanescens*. In blowflies, two pairs of DA-L-IR neurons occur in the lateral rim of the posterior suboesophageal zone (Nässel and Elekes, 1992). They have not been described for *D. melanogaster* (Budnik and White, 1988; Monastirioti, 1999; Mao and Davis, 2009).

Innervation of neuropil areas was sparse: low densities of varicose terminals occur throughout the brain, and only the mushroom body lobes and ventral rim of the brain show higher levels of DA-like immunoreactivity. We did not observe any DA-like immunoreactivity in the optic lobes of *T. evanescens*, similar to *A. mellifera* (Schafer and Rehder, 1989; Schürmann et al., 1989). The mushroom body calyx was also devoid of DA-like immunoreactivity. Similar mushroom body innervation patterns were reported for locusts and blowflies, where all mushroom body areas show DA-like immunoreactivity except the calyces (Nässel and Elekes, 1992; Wendt and Homberg, 1992). Initial reports on DA-like immunoreactivity in *D. melanogaster* only showed innervation of the mushroom body lobes (Zhang et al., 2007), but later studies also discovered DA-L-IR neurites innervating the calyx (Mao and Davis, 2009). *Apis mellifera* show a more pronounced innervation of the mushroom bodies, which consists of many varicose terminals in the calyces and layers of DA-like immunoreactivity in the lobes and pedunculus (Schürmann et al., 1989).

We observed a single DA-L-IR neurite that followed the pedunculus in the direction of the calyx in *T. evanescens*. This neurite resembles the projection of the DA-L-IR neuron cluster lateral to the lateral calyx in *A. mellifera* (Schafer and Rehder, 1989). This projection runs to the medial side of the pedunculus, innervates the vertical lobe, and projects medioventrally along the dorsal side of the medial lobe towards the brain midline. The neurite does not resemble any of the DA-L-IR neurites described in *D. melanogaster* (Claridge-Chang et al., 2009; Krashes et al., 2009; Mao and Davis, 2009; Aso et al., 2010; Aso et al., 2012; Burke et al., 2012).

The most pronounced DA-like immunoreactivity was found at the base of the mushroom body calyx, where a bundle of DA-L-IR fibres follows the lateral rim of the brain, anterior to the mushroom body pedunculus and medial to the calyx. It does not resemble the layered pattern of DA-like immunoreactivity that has been described for the mushroom body pedunculus and lobes of *A. mellifera* (Schürmann et al., 1989), because the bundle projects in the direction of (but could not be observed to innervate) the lobula instead of being restricted to the mushroom bodies. Despite the close location to cluster DA-5, we could not observe a connection between the bundle of DA-L-IR fibres and cluster DA-5, nor with the neurite that projects along the pedunculus. The origin, function or resemblance to other insects is therefore unknown.

**References**

Aso Y, Herb A, Ogueta M, Siwanowicz I, Templier T, Friedrich AB, Ito K, Scholz H, Tanimoto H. 2012. Three dopamine pathways induce aversive odor memories with different stability. PLoS Genet 8(7):e1002768.

Aso Y, Siwanowicz I, Bracker L, Ito K, Kitamoto T, Tanimoto H. 2010. Specific dopaminergic neurons for the formation of labile aversive memory. Curr Biol 20(16):1445-1451.

Bleeker MAK, Van der Zee B, Smid HM. 2006. Octopamine-like immunoreactivity in the brain and suboesophageal ganglion of two parasitic wasps, *Cotesia glomerata* and *Cotesia rubecula*. Anim Biol 56(2):247-257.

Blenau W, Thamm M. 2011. Distribution of serotonin (5-HT) and its receptors in the insect brain with focus on the mushroom bodies: lessons from *Drosophila melanogaster* and *Apis mellifera*. Arthropod Struct Dev 40(5):381-394.

Budnik V, White K. 1988. Catecholamine-containing neurons in *Drosophila melanogaster:* distribution and development. J Comp Neurol 268(3):400-413.

Burke CJ, Huetteroth W, Owald D, Perisse E, Krashes MJ, Das G, Gohl D, Silies M, Certel S, Waddell S. 2012. Layered reward signalling through octopamine and dopamine in *Drosophila*. Nature 492(7429):433-437.

Busch S, Selcho M, Ito K, Tanimoto H. 2009. A map of octopaminergic neurons in the *Drosophila* brain. J Comp Neurol 513(6):643-667.

Claridge-Chang A, Roorda RD, Vrontou E, Sjulson L, Li H, Hirsh J, Miesenbock G. 2009. Writing memories with light-addressable reinforcement circuitry. Cell 139(2):405-415.

Dacks AM, Christensen TA, Agricola HJ, Wollweber L, Hildebrand JG. 2005. Octopamine-immunoreactive neurons in the brain and subesophageal ganglion of the hawkmoth *Manduca sexta*. J Comp Neurol 488(3):255-268.

Dacks AM, Christensen TA, Hildebrand JG. 2006. Phylogeny of a serotonin-immunoreactive neuron in the primary olfactory center of the insect brain. J Comp Neurol 498(6):727-746.

Farris SM, Schulmeister S. 2011. Parasitoidism, not sociality, is associated with the evolution of elaborate mushroom bodies in the brains of hymenopteran insects. Proc Biol Sci 278(1707):940-951.

Fischer S, Muller CH, Meyer-Rochow VB. 2011. How small can small be: the compound eye of the parasitoid wasp *Trichogramma evanescens* (Westwood, 1833) (Hymenoptera, Hexapoda), an insect of 0.3- to 0.4-mm total body size. Vis Neurosci 28(4):295-308.

Gregory TR. 2001. Coincidence, coevolution, or causation? DNA content, cell size, and the C-value enigma. Biol Rev 76(1):65-101.

Hammer M, Menzel R. 1995. Learning and memory in the honeybee. J Neurosci 15(3 Pt 1):1617-1630.

Haverkamp A, Smid HM. 2014. Octopamine-like immunoreactive neurons in the brain and subesophageal ganglion of the parasitic wasps *Nasonia vitripennis* and *N. giraulti*. Cell Tissue Res 358(2):313-329.

Hoyer SC, Liebig J, Rossler W. 2005. Biogenic amines in the ponerine ant *Harpegnathos saltator*: serotonin and dopamine immunoreactivity in the brain. Arthropod Struct Dev 34(4):429-440.

Krashes MJ, DasGupta S, Vreede A, White B, Armstrong JD, Waddell S. 2009. A neural dircuit mechanism integrating motivational state with memory expression in *Drosophila*. Cell 139(2):416-427.

Kreissl S, Eichmuller S, Bicker G, Rapus J, Eckert M. 1994. Octopamine-like immunoreactivity in the brain and subesophageal ganglion of the honeybee. J Comp Neurol 348(4):583-595.

Liu C, Placais PY, Yamagata N, Pfeiffer BD, Aso Y, Friedrich AB, Siwanowicz I, Rubin GM, Preat T, Tanimoto H. 2012. A subset of dopamine neurons signals reward for odour memory in *Drosophila*. Nature 488(7412):512-+.

Makarova AA, Polilov AA. 2013. Peculiarities of the brain organization and fine structure in small insects related to miniaturization. 2. The smallest Hymenoptera (Mymaridae, Trichogrammatidae). Entomol Rev 93(6):714-724.

Mao Z, Davis RL. 2009. Eight different types of dopaminergic neurons innervate the *Drosophila* mushroom body neuropil: anatomical and physiological heterogeneity. Front Neural Circuits 3:5.

Mares S, Ash L, Gronenberg W. 2005. Brain allometry in bumblebee and honey bee workers. Brain Behav Evol 66(1):50-61.

Menzel R, Giurfa M. 2001. Cognitive architecture of a mini-brain: the honeybee. Trends Cogn Sci 5(2):62-71.

Monastirioti M. 1999. Biogenic amine systems in the fruit fly *Drosophila melanogaster*. Microsc Res Tech 45(2):106-121.

Nässel DR. 1988. Serotonin and serotonin-immunoreactive neurons in the nervous system of insects. Prog Neurobiol 30(1):1-85.

Nässel DR, Elekes K. 1992. Aminergic neurons in the brain of blowflies and *Drosophila*: dopamine- and tyrosine hydroxylase-immunoreactive neurons and their relationship with putative histaminergie neurons. Cell Tissue Res 267.

Nässel DR, Meyer EP, Klemm N. 1985. Mapping and ultrastructure of serotonin-immunoreactive neurons in the optic lobes of three insect species. J Comp Neurol 232(2):190-204.

Niven JE, Farris SM. 2012. Miniaturization of nervous systems and neurons. Curr Biol 22(9):R323-329.

Paulk A, Sean Millard S, Van Swinderen B. 2013. Vision in *Drosophila*: Seeing the world through a model's eyes. Annu Rev Entomol. Vol 58. p 313-332.

Polilov AA. 2005. Anatomy of the feather-winged beetles *Acrotrichis montandoni* and *Ptilium myrmecophilum* (Coleoptera, Ptiliidae). Zoologichesky Zhurnal 84(2):181-189.

Polilov AA. 2012. The smallest insects evolve anucleate neurons. Arthropod Struct Dev 41(1):29-34.

Polilov AA. 2015. Small is beautiful: features of the smallest insects and limits to miniaturization. Annu Rev Entomol 60(1):103-121.

Rehder V, Bicker G, Hammer M. 1987. Serotonin-immunoreactive neurons in the antennal lobes and suboesophageal ganglion of the honeybee. Cell Tissue Res 247(1):59-66.

Schafer S, Rehder V. 1989. Dopamine-like immunoreactivity in the brain and suboesophageal ganglion of the honeybee. J Comp Neurol 280(1):43-58.

Schroter U, Malun D, Menzel R. 2007. Innervation pattern of suboesophageal ventral unpaired median neurones in the honeybee brain. Cell Tissue Res 327(3):647-667.

Schürmann FW, Elekes K, Geffard M. 1989. Dopamine-like immunoreactivity in the bee brain. Cell Tissue Res 256(2):399-410.

Schürmann FW, Klemm N. 1984. Serotonin-immunoreactive neurons in the brain of the honeybee. The Journal of Comparative Neurology 225(4):570-580.

Seidel C, Bicker G. 1996. The developmental expression of serotonin-immunoreactivity in the brain of the pupal honeybee. Tissue Cell 28(6):663-672.

Sinakevitch I, Niwa M, Strausfeld NJ. 2005. Octopamine-like immunoreactivity in the honey bee and cockroach: comparable organization in the brain and subesophageal ganglion. J Comp Neurol 488(3):233-254.

Sinakevitch I, Strausfeld NJ. 2006. Comparison of octopamine-like immunoreactivity in the brains of the fruit fly and blow fly. J Comp Neurol 494(3):460-475.

Sitaraman D, Zars M, LaFerriere H, Chen YC, Sable-Smith A, Kitamoto T, Rottinghaus GE, Zars T. 2008. Serotonin is necessary for place memory in *Drosophila*. Proc Natl Acad Sci U S A 105(14):5579-5584.

Srinivasan MV. 2010. Honey bees as a model for vision, perception, and cognition. Annu Rev Entomol 55:267-284.

Van der Woude E, Smid HM. 2017. Effects of isometric brain-body size scaling on the complexity of monoaminergic neuron networks in a minute parasitic wasp. Brain Behav Evol. *In press*.

Van der Woude E, Smid HM, Chittka L, Huigens ME. 2013. Breaking Haller's rule: brain-body size isometry in a minute parasitic wasp. Brain Behav Evol 81(2):86-92.

Waddell S. 2013. Reinforcement signalling in *Drosophila*; dopamine does it all after all. Curr Opin Neurobiol 23(3):324-329.

Wendt B, Homberg U. 1992. Immunocytochemistry of Dopamine in the brain of the locust *Schistocerca gregaria*. J Comp Neurol 321(3):387-403.

Whitfield JB. 2003. Phylogenetic insights into the evolution of parasitism in Hymenoptera. Adv Parasitol. Vol Volume 54: Academic Press. p 69-100.

Zhang K, Guo JZ, Peng Y, Xi W, Guo A. 2007. Dopamine-mushroom body circuit regulates saliency-based decision-making in *Drosophila*. Science 316(5833):1901-1904.
